# Epidemiology, Biotic Interactions and Biological Control of Armillarioids in the Northern Hemisphere

**DOI:** 10.3390/pathogens10010076

**Published:** 2021-01-16

**Authors:** Orsolya Kedves, Danish Shahab, Simang Champramary, Liqiong Chen, Boris Indic, Bettina Bóka, Viktor Dávid Nagy, Csaba Vágvölgyi, László Kredics, György Sipos

**Affiliations:** 1 Department of Microbiology, Faculty of Science and Informatics, University of Szeged, Szeged, Közép fasor 52, H-6726 Szeged, Hungary; kedvesorsolya91@gmail.com (O.K.); danish18581@yahoo.co.in (D.S.); simang5c@uni-sopron.hu (S.C.); liqiongchen2016@163.com (L.C.); boka.tina@gmail.com (B.B.); viktor.david.nagy@gmail.com (V.D.N.); csaba@bio.u-szeged.hu (C.V.); 2 Functional Genomics and Bioinformatics Group, Research Center for Forestry and Wood Industry, University of Sopron, Bajcsy-Zsilinszky str. 4., H-9400 Sopron, Hungary; boris.indjic@phd.uni-sopron.hu

**Keywords:** *Armillaria*, biocontrol, epidemiology, management

## Abstract

Armillarioids, including the genera *Armillaria*, *Desarmillaria* and *Guyanagaster*, represent white-rot specific fungal saprotrophs with soilborne pathogenic potentials on woody hosts. They propagate in the soil by root-like rhizomorphs, connecting between susceptible root sections of their hosts, and often forming extended colonies in native forests. Pathogenic abilities of *Armillaria* and *Desarmillaria* genets can readily manifest in compromised hosts, or hosts with full vigour can be invaded by virulent mycelia when exposed to a larger number of newly formed genets. Armillaria root rot-related symptoms are indicators of ecological imbalances in native forests and plantations at the rhizosphere levels, often related to abiotic environmental threats, and most likely unfavourable changes in the microbiome compositions in the interactive zone of the roots. The less-studied biotic impacts that contribute to armillarioid host infection include fungi and insects, as well as forest conditions. On the other hand, negative biotic impactors, like bacterial communities, antagonistic fungi, nematodes and plant-derived substances may find applications in the environment-friendly, biological control of armillarioid root diseases, which can be used instead of, or in combination with the classical, but frequently problematic silvicultural and chemical control measures.

## 1. Introduction

The armillarioid genera *Armillaria* and *Desarmillaria* are among the most important fungal plant pathogens causing a root disease that has long been recognized as a severe ecological and economical threat worldwide. Armillarioid species are well-known white-rot-specific wood-decaying fungi [1]. They target hundreds of tree species and woody shrubs, and affect several million hectares of forests, commercial orchards, vineyards, as well as trees in urban areas (e.g., parks, gardens) in all boreal, temperate, and tropical regions [2,3,4,5,6,7,8,9,10,11]. Before tree mortality, colonization may lead to crown dieback, lower-stem deformation, resinous-root lesion, down-wood accumulation, and growth reduction in several tree species [12,13,14,15]. Most armillarioid species can inherently survive for decades in infected stumps and parts of their root system due to their vigourous and persistent nature. This long persistence in susceptible plant tissues may then cause serious damage in the developmental process of plants. Severe losses can occur in coniferous and deciduous trees, orchards, or vineyards if planting occurs in infected soils [16,17,18]. The majority of armillarioid species, acting as facultative necrotrophs, are primary pathogens carrying an innate infectious potential in colonizing living hosts, while others are considered as opportunistic or “weak” pathogens invading already compromised trees. The observed virulence of pathogenic species depends on the individual infectious abilities, host species, age of the tree and influence of the environment [9,10,19,20,21,22,23,24].

This review aims to provide an overview about the epidemiology of pathogenic armillarioid species, their infection process, the biotic impacts on their pathogenesis, as well as the available and potential biocontrol options to manage the problems caused by them, with particular emphasis on the role of molecular biology tools with the potential to aid the fight against armillarioid root rot diseases.

## 2. Identification

**Figure 1:**
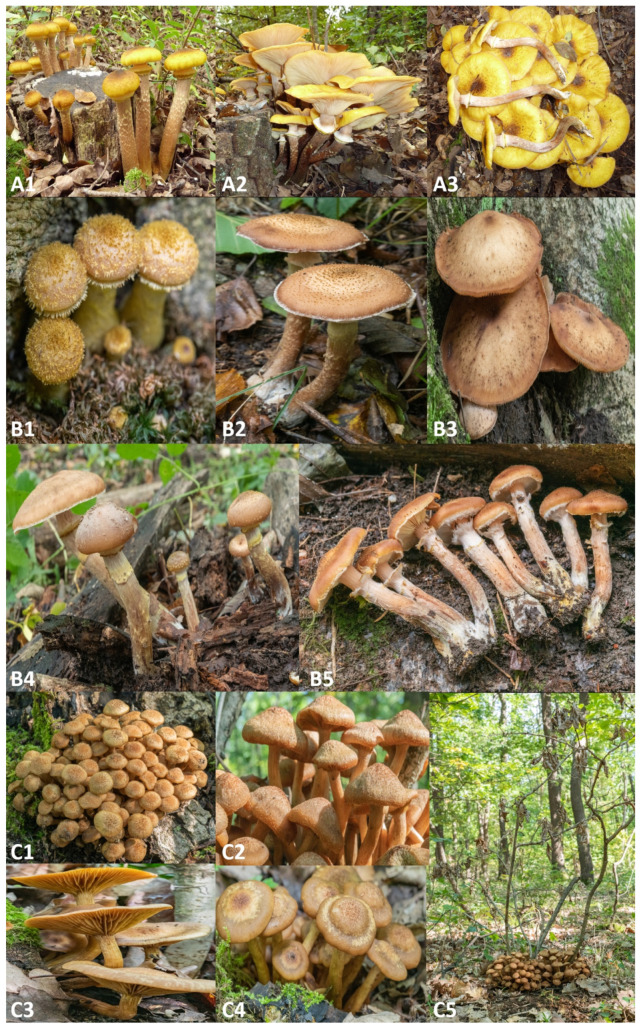
The prominent *Armillaria* and *Desarmillaria* species from broad-leaved forests in Central Europe: *Armillaria mellea* (**A1**–**A3**); frequent in Turkey oak and pedunculate oak forests (Keszthely Hills, Hungary); *Armillaria gallica* (**B1**–**B5**); (**B1**,**B2)** representing freshly grown, while **B3** already decaying fruiting bodies; some were found growing out from the base of still healthy-looking beech trees (**B1**–**B3**), others (**B4**) were prevalently covering already rotten forest logs and woody fragments; *Desarmillaria tabescens* (**C1**–**C5**); frequently found in oak forests during the first weeks in autumn, in some cases (**C5**) fruiting bodies were also observed growing right from the base of dead little oak trees (Sopron Hills, Hungary).

Morphological characteristics such as the presence of annulus, the structure of stipe and velar remnants, pileus colour and ornamentation, and the colour and contexture of the scales on the pileus are informative for the delineation of various *Armillaria* and *Desarmillaria* species. As obvious observable traits, *Armillaria* species have annulated fruiting bodies and produce abundant rhizomorphs (root-like, dark mycelial strings), whereas *Desarmillaria* species are exannulated and lack rhizomorphs under field conditions (Figure 1). The branching of rhizomorphs, being either monopodial or dichotomous, may also indicate possible species identities, as far as *A. ostoyae*, *A. mellea*, *A. borealis* and *A. calvescens* produce dichotomous, while *A. gallica*, *A. cepistipes* and *A. hinnulea* make monopodial filaments [25,26].

The species identities of *Armillaria* isolates can be confirmed by using species-specific haploid tester strains in diploid–haploid pairing assays [27,28,29,30]. Then the clonal individuals or genets can be identified in further pairing assays by testing the self- and non-self-recognition through intraspecific somatic incompatibility reactions [27,28,31,32]. Although the efficiency and reliability of the diploid–haploid pairing tests is often debated [1], using well-maintained and genetically characterized haploids combined with proper controls may still offer a clear practical benefit in defining the “biological” species identities from various field isolates.

In the near past, biochemical tools like isoenzyme analysis [33,34] or the application of monoclonal and polyclonal antibodies [35] might have also helped in *Armillaria* identification. Bragaloni et al. [33] analysed the isozyme profiles of European *Armillaria* species for identification purposes. Esterase (E.C. 3.1.1.1.), glutamic-oxalacetic transaminase (2.6.1.1.), phosphoglucomutase (E.C. 2.7.5.1.), alcohol dehydrogenase (E.C. 1.1.1.1.), and polygalacturonase (E.C. 3.2.1.15.) enzyme profiles proved to be complex enough for proper identification [33]. Bruhn [34] investigated mycelial growth characteristics as well as esterase and polyphenol oxidase production of *A. mellea*, *D. tabescens,* and *A. gallica*. For more closely related species, this method could be a more challenging option [36].

Nowadays, when numerous genomes are already available, modern molecular-based identification techniques/methods, such as qualitative PCR, quantitative real-time PCR or PCR-DGGE are being routinely used for the detection of phytopathogenic and antagonistic fungi [37,38,39,40,41]. The application of molecular markers introduced a new era of species identification. Restriction fragment length polymorphism (RFLP) analysis of the internal transcribed spacer (ITS) and intergenic spacer (IGS) regions of the rDNA [36,42,43,44,45,46,47,48,49,50,51,52], the nuclear rRNA [37,51], or the nuclear DNA [52,53] proved to be useful during early studies aimed at *Armillaria* identification. Harrington and Wingfield [54] amplified the intergenic spacer (IGS) of the ribosomal RNA gene cluster with polymerase chain reaction (PCR) and digested the amplified products with the restriction enzymes *Alu*I and *Nde*I, *Bsm*I or *Hind*II. Each examined taxon (*A. borealis*, *A. calvescens*, *A. cepistipes*, *A. gallica*, *A. gemina*, *A. mellea*, *A. solidipes/A. ostoyae*, *A. sinapina* and *D. tabescens*) could be distinguished by their polymorphisms after these restrictions, suggesting that it is a rapid and cost-effective option for *Armillaria* identification. Mitochondrial DNA analysis, DNA-DNA hybridization and the random amplified polymorphic DNA (RAPD) method may also give useful information for the identification of *Armillaria* species; however, the most rapid methods are based on the amplification and sequence analysis of conserved rDNA regions [36] like the internal transcribed spacer (ITS) region of the ribosomal RNA gene cluster (ITS1 and ITS2) or the intergenic spacer region IGS-1 [45,55,56,57,58]. Nevertheless, rDNA sequence data do not confirm the differences between closely related species of *Armillaria* [36,57], therefore many authors found a nuclear gene, the translation elongation factor 1α (*tef1*) more suitable than ITS sequence analysis for the identification of differences between *Armillaria* species [1,59,60,61,62,63]. The product of this gene is transporting amino-acyl tRNAs to the ribosomes and plays a role in eukaryotic protein synthesis [64]. The diagnostic assay based on partial sequences of the *tef1* gene can be utilized for the identification of closely related *Armillaria* species without the need for RFLPs and the subsequent interpretation of banding patterns [59,61,63,65,66,67,68,69]. Brazee et al. [65] used partial sequences of *tef1*, RNA polymerase II (*rpb2*) and the nuclear large subunit (nLSU) genes for identification. *Tef1* was the only gene which could differentiate between all 6 examined species (*A. calvescens*, *A. gallica*, *A. gemina*, *A. mellea*, *A. sinapina* and *A. solidipes*) and revealed differences between the closely related species *A. calvescens* and *A. gallica*. The limitation of these techniques is the necessity of pure culture and clean mycelium. The development of specific PCR methods could be a solution to this limitation [38,63], as such tools can be optimized also for infected plant materials.

**Figure 2:**
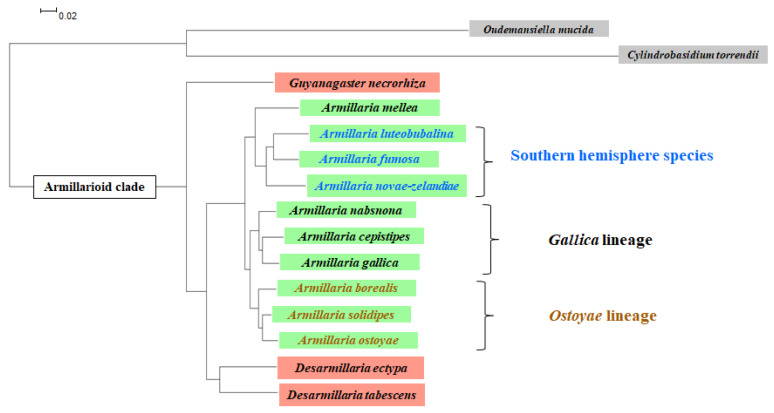
Genome-level phylogram of orthologous proteins from the Armillarioid clade. Orthofinder [75] predicted 17,376 orthogroups using the protein sequences from all the 15 taxa. MAFFT [76] was then applied to build the multiple sequence alignment, and the maximum-likelihood phylogenetic tree was inferred using FastTree [77]. *Guyanagaster*, *Desarmillaria* and *Armillaria* constitute separate genera within the Armillarioid clade, whereas *Oudemansiella mucida* and *Cylindrobasidium torrendii* represent the closest outgroup taxa.

So far, defining species or especially intraspecies boundaries for various *Armillaria* isolates, often coming from different continents, has been a challenging task [1]. The use of highly conserved markers (ITS, IGS, *tef1*, etc.), either individually or in combination, does not always provide the required resolution for safely identifying new species and establishing reliable clustering of all possible interspecies clades [70,71,72]. Here we propose a new, genome level approach for creating a phylogenetic tree by using the power of comparative genomics, based on the full spectrum of orthologues from available genomes of interest [73] (Champramary et al. ms in prep) (Figure 2). Our current data confirm the previously suggested armillarioid clade (*Physalacriaceae*, *Basidiomycota*) comprising of three genera, namely *Armillaria*, *Desarmillaria* and *Guyanagaster* [74]. The genera *Desarmillaria* and *Guyanagaster*, both consisting of two known species so far, represent extant taxa of early armillarioids, while *Armillaria* species and lineages are mostly pathogenic wood-decaying fungi that recently diverged. Furthermore, the genome-level analysis of orthologues approves the previous findings of *tef1* sequence analysis in predicting several *Armillaria* lineages and separating European *A. ostoyae* and North American *A. solidipes* isolates (the latter ones frequently reported in the literature as *A. ostoyae* but referred further in this article as *A. solidipes*) at the species level [70,71] (Figure 2).

## 3. Biodiversity, Population Genetics

*Armillaria* species are soilborne pathogens that use root-like rhizomorphs as persistent propagative structures in the soil and to colonize roots and fallen logs. The networks of rhizomorphs represent territorially expanding, often vastly extended and long-lived, clonal individuals or genets [78]. Population-level studies corroborated that genets of the same species maintain discrete territories, possibly by the prevalence of somatic incompatibility between adjacent colonies [79]. Genets of different species, representing either apparently non-competing saprotrophic (*A. cepistipes*) and pathogenic (*A. ostoyae*) species, or others (*A. altimontana* and *A. solidipes*) showing signs of competitive in situ exclusion, may occur sympatrically with significant spatial overlaps between them [80,81]. For the evaluation of the genetic structures of two spatially distinct *A. cepistipes* populations, neutral genetic traits as single sequence repeats (SSR) and single-nucleotide polymorphisms (SNPs) have proven to offer distinctive tracking of inherent genetic variabilities either within indigenous (50–100 km) or between distant (1000 km) fungal populations [82]. In contrast to the prevalence of extended genets in native forests, a newly established ornamental landscape with trees infected by *A. mellea* mycelia, representing a high proportion of unique genotypes in single trees, suggested a significant role of local spore dispersal in *Armillaria*-related ecology [83].

## 4. Distribution and Host Range of Pathogenic Armillarioid Species and Lineages 

*Armillaria* root disease is a plant disease of varying geographic distribution and virulence, with a broad spectrum of hosts. In the Northern Hemisphere it has been frequently reported from Europe [9,18,25,71,84,85,86,87,88,89,90], Africa [71,91,92,93], Asia [71,94,95,96,97] and America [10,24,25,71,98,99,100,101,102]. There has been a long history of great interest in exploring the ecology of *Armillaria* species in plantations, managed forests, or natural re-generations in different countries throughout the Northern Hemisphere. The distribution of *Armillaria* species varies, generally based on the tree species, as well as on their stumps or dead substrates. Most of the *Armillaria* species show preference towards either coniferous or broad-leaved forest environments [9,100,101,102,103,104,105]. Although native coniferous forests in the Northern Hemisphere are predominantly inhabited by *A. cepistipes* and *A. ostoyae*, various oak (*Quercus* spp.) and other broad-leaved species are mostly exposed to *A. mellea*, *A. gallica* and *D. tabescens* (Figure 1) [106]. These five most common armillarioid species differ in virulence, geographical distribution and host range. 

Currently, inferences from our orthogroup-based phylogenetic data (Figure 2), combined also with previous large-scale statistical analysis of *tef1* sequences [70], support the confinement of possible “Ostoyae”, “Mellea” and “Gallica” lineages. The species of the “Ostoyae” lineage, besides *A. ostoyae* and *A. solidipes*—likely representing primary pathogens—may potentially include *A. gemina*, *A. borealis* and *A. sinapina*. In Europe, *A. ostoyae* (Supplementary Table S1) was recorded from many countries, including Switzerland [107], Ukraine [108] and the southern mountains of Serbia at 800–1800 m [36]. In Albania, *A. ostoyae* was common, causing significant damage on black pine *(Pinus nigra*), Scots pine (*Pinus sylvestris*), Norway spruce (*Picea abies*), Serbian spruce (*Picea omorika*) and silver fir (*Abies alba*) at altitudes from 600 to 1800 m, while at lower altitudes (ca. 100–800 m), *A. ostoyae* was recorded on maritime pine (*Pinus pinaster*), Mediterranean *cypress (Cupressus sempervirens*) and common juniper (*Juniperus communis*) [9]. The species was also reported from England [109,110], while Greece [111] appears to be the southernmost limits of its distribution in the Balkan Peninsula, whereas in Italy the distribution extends to Calabria, the southernmost part of the peninsula [112]. *Armillaria solidipes* is widely distributed in coniferous forests of Canada (Ontario, British Columbia) as well as the North-Western, interior South-Western, North-Central and North-Eastern USA (Supplementary Table S2) [113]. Within the Western USA it has been commonly found in the pacific North-West (Northern Idaho, Western Montana, Oregon and Washington) and the Colorado Plateau. *Armillaria sinapina* (Supplementary Table S3) is considered as a weak pathogen of diverse hosts [114], it has been reported from a variety of conifer—white spruce (*Picea glauca*), mountain hemlock (*Tsuga mertensiana*)—and hardwood—birch (*Betula* spp.), trembling aspen (*Populus tremuloides*), willow (*Salix* spp.)—forest trees on sites with diverse climates in Alaska [115], as well as from Douglas-fir (*Pseudotsuga menziesii*), western hemlock (*Tsuga heterophylla*) and western redcedar (*Thuja plicata*) in the southern interior of British Columbia [102]. The host range of *A. gemina* (Supplementary Table S4) was found to be restricted to sugar maple (*Acer saccharum*) in Canada [116]. This species has also been reported in the Eastern USA (Vermont and New York) on sugar maple, American beech (*Fagus grandifolia*) and yellow birch (*Betula alleghaniensis*) [117]. *Armillaria borealis* (Supplementary Table S5) has the most Northern distribution among the European armillarioid species, its limit coinciding with the limit of woody vegetation in Scandinavia (Supplementary Table S3) [118]. *A. borealis* is known as a secondary pathogen of weakened coniferous and deciduous trees and its aggressive behaviour is rare [119]. The record of *A. borealis* from Albania represents the southernmost observation in Europe; with the nearest record being from Slovenia [120]. 

As a member of another possible lineage, *Armillaria mellea* (Supplementary Table S6, Figure 1(A1–A3), Figure 2) is the most common organism causing *Armillaria* root rot disease, which affects a wide range of more than 500 host species, including ornamentals, forest trees (coniferous and broad-leaved ones), cultivated woody plants, grapevine (*Vitis vinifera*) and fruit trees [9,18,19,83,111,121,122,123,124,125]. *A. mellea* is known to occur from England [109,110] to Central, Southern and Western Europe [126,127], as well as in North America, mainly in broad-leaved, less commonly in coniferous forests [3,128]. *A. mellea* is the most predominant armillarioid species in Greece [111,129]. In peach (*Prunus persica*) orchards in the South-Eastern USA the disease is caused primarily by *D. tabescens* and *A. mellea* [123,130]. 

The “Gallica” lineage (Figure 2) seems to represent opportunistic or weak pathogenic species. These include *A. gallica*, *A cepistipes*, *A. calvescens*, *A. nabsnona*, *A. altimontana* (formerly NABS X) and the so far unnamed “Nag. E” isolates from Japan [61,70,71]. *Armillaria gallica* (Supplementary Table S7, Figure 1(B1–B5)) occurs in England [109,110], as well as continental Europe [108,122,131], while in North America it is commonly reported east of the Rocky Mountains and in West Coast states of the USA [132], Arizona [133], and it is also known from Mexico [134] and Japan [95]. *A. gallica* is known to share many forest types in common with *A. mellea*, e.g., it was reported that *A. mellea* and *A. gallica* had overlapping geographic ranges in Central North America, the main reason of which probably lies in both of the two species favouring similar hosts, especially various oak and broad-leaved plant species [132,134]. Interestingly, past surveys have noted that besides *A. mellea*, *A. gallica* can be highly aggressive and become a major threat to forest decline. Under stress such as drought condition and climate change, *A. gallica* turned out as an aggressive pathogen on several new hosts of Methly plum (*Prunus salinica*), Monterey pine (*Pinus radiata*) and loblolly pine (*Pinus taeda*) in the island of Hawaii [135]. However, very interestingly, current plant disease reports highlight *A. gallica* isolates from Central Mexico acting as virulent pathogens on living trees [136], which may well be in line with previous data on *A. gallica* virulently invading peach trees also in Mexico [98]. The possible switch from opportunistic pathogenicity towards primary necrotrophy could be related to cryptic speciation events within the Mexican *A. gallica* population. The preferentially saprotrophic *A. cepistipes* (Supplementary Table S8) is the most common armillarioid species in Europe with a wide distribution from Northern Europe through Switzerland [87] to Ukraine [108], Central Albania, Northern Greece [111], and the southernmost part of Italy [112]. This species was occasionally found to cause disease on grapevines (*Vitis* spp.) at altitudes ranging from 800 to 1800 m, mostly as a saprophyte on conifers and broad-leaved trees in beech and silver fir forests [9]. *A. cepistipes* and *A. ostoyae* often occur in the same forest types, e.g., in Serbia they were observed together in the cold-tolerant conifer forest type dominated by silver fir and Norway spruce [106]. *Armillaria calvescens* (Supplementary Table S9) is mostly restricted to northern hardwood (beech–birch–maple) forests in North-Eastern North America [100]. *A. nabsnona* was found on several hardwood tree species in the continental USA—bigleaf maple (*Acer macrophyllum*), vine maple (*Acer circinatum*), red alder (*Alnus rubra*), Sitka spruce (*Picea sitchensis*), western balsam poplar (*Populus trichocarpa*) and western hemlock [137]; Hawaii—‘Ohi’a lehua (*Metrosideros polymorpha*), Nepalese alder (*Alnus nepalensis*) and Chinese banyan (*Ficus microcarpa*) [58], as well as Hokkaido, Japan—Mongolian oak (*Quercus mongolica* var. *grosseserrata*) [138,139], while *A. altimontana* has been found on hardwoods (elder species) and conifers—grand fir (*Abies grandis*), western white pine (*Pinus monticola*)—in the conifer forest zone of western interior North America [81,140], where it is frequently co-occurring with *A. solidipes*.

*Desarmillaria tabescens* (Supplementary Table S10, Figure 1(C1–C5)) is considered as a typical saprotroph with a world-wide distribution and a wide host range [141]; however, this species may also be a primary pathogen, as it has been observed in blue gum (*Eucalyptus* spp.) introduced to South-West France [131], in peach [142], or as an opportunistic parasite in cork oak (*Quercus suber*) [131]. *D. tabescens* is usually found in oak-dominated forests [106] and observed mostly on butts and root systems of dead or dying trees and on stumps. It has a southern distribution, but in the maritime climate of Western Europe its distribution area extends to Southern Britain [109]. In Albania it was found frequently on several species of oak and poplar (*Populus* spp.), as well as on blue gum [9]. *D. tabescens* occasionally also caused disease on pear (*Pyrus* spp.) and almond (*Prunus dulcis*) trees. In Serbia, this species was found only at altitudes below 550 m [106], but in Greece and Albania it also occurred at higher altitudes, up to 1150 and 1300 m, respectively [9,111]. This difference may be related with climatic conditions, as the climate of Serbia is more continental than that of Greece or Albania. In the South-Eastern USA, *Armillaria* root rot on peach is caused primarily by *D. tabescens* [142].

## 5. Biology and Infection Strategies of Armillarioid Species

Transitions between exploratory rhizomorphs and reproductive structures or adjusting to the environment for nutrient acquisition through specialized mycelia and hyphae, either with saprotrophic or necrotrophic activities, require complex morphological and functional changes [73,143,144]. Current genomic and transcriptomic studies demonstrated that gene expression profiles from rhizomorphs of *A. ostoyae* represent evolutionarily recent *Armillaria* lineage-specific gene sets, and rhizomorphs indeed share patterns of upregulated genes and their cis-regulatory elements with that of the fruiting bodies, indicating possible morphogenetic relationship between them [73]. So far, more than 10 *Armillaria* genomes—including also recent genome-level updates—have been released [73,82,119,145,146,147,148,149]. Comparative genomic studies of 4 *Armillaria* species revealed a full complement of plant cell-wall-degrading enzymes and pathogenicity-related genes, including also genes involved in chitin-binding and others in pectinolytic activities. Current research interests are focused on setting up well-controlled inoculation tests to identify which mechanisms and molecular factors drive rhizomorph contact, penetration, and hyphal-host communication during the progress of infection [150]. Defining genomic and transcriptomic differences between pathogenic and saprotrophic activities, and also between virulent and non-virulent isolates of pathogenic species, and their interactions with the host-associated microbial communities may further help us to understand the complex interactive networks between invasive fungal mycelia and the host.

### 5.1. Transmission and Infection Pathways

Armillarioid species spread through an underground dispersal mechanism either via their rhizomorphs or through physical root connections. When the roots of infected plants meet uninfected roots of adjacent and susceptible hosts, they form a disease centre, which may be limited to a few trees or spread over several hectares in a forest, orchard or vineyard. A disease centre is usually occupied by one or more diploid individuals which may be derived from the former forest stands [132,151]. Low genotypic diversity of armillarioid species detected in most disease centres entirely unveiled the destructive impacts from these short distant infection processes. In the Landes de Gascogne forest of France, which is the largest monospecific maritime pine plantation forest in Europe, only one genotype or one predominated genotype was detected for *A. ostoyae* in most disease centres [152,153]. In Northern Turkey, as multilocus genotyping indicated, a single genet of *A. ostoyae*, at least 0.2 ha in size, was identified from the disease centre associated with the dying 60-year-old Scots pines in a naturally regenerated forest [154].

So as to become an efficient plant invader, armillarioids undergo complex developmental changes to colonize plant tissues and complete their life cycle [155]. Rhizomorphs conquer the infected host tissues by deploying hyphae for nutrient acquisition and also extend into the soil to travel and encompass further susceptible hosts [156]. Networks of rhizomorphs can breach mechanical obstacles and may function as an organ system where absorption and transportation occur and facilitate underground spread [157,158,159]. These benefits led to the extension of several *Armillaria* species (*A. ostoyae*, *A. gallica* and *A. cepistipes*) over vast territories [13,79,160,161]. Interestingly, *Armillaria* species with dichotomously branched rhizomorphs, such as *A. ostoyae*, *A. mellea* and *A. borealis* were a lot more aggressive in killing seedlings compared with monopodially branched species such as *A. cepistipes*, *A. gallica*, *A. sinapina* and *A. calvescens* [162]. The preferentially saprotrophic, less pathogenic *Armillaria* species propagate their mycelia at higher growth rates by harnessing more abundant and vigourous rhizomorphs in the soil with monopodial branching patterns [26]. These species, rather than causing lethal diseases, prefer to derive nutrition from rotten wood or humus in the soil; and such rhizomorphs, through non-invasive physical contacts, may also share their nutrient resources with potential symbiotic plant partners [163]. Both field observations and laboratory experiments confirmed that rhizomorphs of facultative parasitic species reinforce their foraging efficiency by developing significantly more growth tips to increase their competitiveness in soil when confronted with the larger rhizomorph systems of saprotrophic species [164]. 

Air pores, hydrophobic structures built of hyphae emerging from the mycelial surface, associate with a net of gas channels inside the mycelia and conduct oxygen into rhizomorphs. This intricate system facilitates efficient oxygen diffusion for the aeration and growth of rhizomorphs, therefore very likely contributing to the broader and deeper spread of inoculum into low oxygen environments in the soil and possibly under the bark of the trees [165].

Physical contacts play an essential role in the spread of *D. tabescens* and *A. mellea*, because these two armillarioid species produce much less rhizomorphs in the soil, while others such as *A. gallica* and *A. cepistipes* generally infect through the explorative rhizomorphs [166]. The growth of the most critical mycelial structures, i.e., mycelial fans, and rhizomorphs through the host tissue and surrounding soil has long been thought to be the two main modes of infection and spread of armillarioid root diseases [90]. Besides the parasitic behaviour, the fungus can also persist as a saprophyte in the form of a mycelium, colonizing the dead roots and wood in the soil of vineyards, urban planting areas, orchards and timber plantations. The colonized and infected plant tissue or woody debris in soil serve as a long-term source of inoculum, colonizing and infecting the roots of new-planted trees through physical contact, which also increases the risk of mortality in the next rotation of trees. The saprophytic behaviour enables the fungal inoculum residing inside the roots and wood to persist for many years in a forest stand [167].

As an example, *A. ostoyae* may commonly dominate in the vicinity of pre-existing forest areas, because the remaining forest fragments ensure a reservoir of inoculum for infection of re-established forest plantations; where root fragments, woody debris and small woody plants offer nutrition for the survival [90]. In a maritime pine plantation heavily infested by *A. ostoyae*, the tree mortality significantly increased after planting. From the third year on, besides that the contribution of the primary inoculum still being essential, the newly dead pine trees served as a secondary inoculum and played an escalating role along with time [168]. The mortality rates increased as the distance between the colonized stumps and healthy trees decreased.

Although *Armillaria* fruiting bodies produce vast quantities of basidiospores, often leaving dense local spore prints behind, haploid mycelia germinating from the spores and invading plants appear fairly unobservable in nature. As a likely explanation, germinating basidiospores and haploid mycelia on natural substrates that are not easily accessible to them could either be short-lived or become dormant, and then their genetic survival and contribution to new infectious abilities are much dependent on the possibilities to interact and fuse with another compatible haploid partner [169]. The formation of diploids and haploid mosaic cells can significantly increase phenotypic plasticity in accessing natural resources and adapting to new host environments [170,171]. In fact, in an outdoor inoculation experiment, haploid *A. ostoyae* isolates were unable to invade seedlings and saplings of Norway spruce, and only diploid mycelia could be recovered from the infected plants, indicating that the colonization of live plant tissues was readily conditioned on a prior onsite diploidization event [169]. There is also evidence that new genotypes from basidiospores would favour colonization on clear-cutting or the planting of new conifer stands. In a newly set and disturbed forest environment, *A. ostoyae* became highly pathogenic due to the large number of distinct diploid genets created most likely by the actual spread of basidiospores [172]. Under native undisturbed forest conditions, the epidemiological importance of basidiospores can be detected and tracked by adjusting to the proper spatial scale for sampling, within and between populations, and then relying on the population genetic analyses of genets. It has been shown that sexual spore dispersal may be more efficient at fair spatial scales, for example, a few kilometres in contrast to larger spatial distances [83,173].

### 5.2. Penetration, Colonization and Disease Development

*Armillaria* species may act either as a primary pathogen frequently observed in disease centres causing gradual, multiyear reduction on the growth and yield of healthy trees, or as a secondary pathogen, infecting and killing already weakened trees [174]. When colonizing living hosts, rhizomorphs penetrate the root surface by combining mechanical pressure and enzymatic activities. Then the penetrating rhizomorphs form mycelial fans underneath the bark, induce tissue necrosis and decompose the underlying cambium, causing the decay of the secondary xylem [22].

At the initiation of the colonization and during the invasion of plant tissues, plant cell wall degrading enzymes and pathogenicity factors are secreted into the interacting fungal exudates. Along with host metabolites, these exudates play significant roles in driving the interactive processes of pathogenesis [102,175,176]. The spectrum of enzymes encoded in *Armillaria* genomes ensures the potential for efficient depolymerization and mineralization of all plant cell wall biopolymers, including lignin, pectin, cellulose, and hemicellulose [73]. Evidence from the transcriptome of a mycelial fan of *A. solidipes* isolated from a naturally infected tree confirmed, that under native conditions, *Armillaria* expresses an array of genes encoding enzymes required for the breakdown of plant cell wall components [59].

*Armillaria* species differ in their rhizomorphs, the rate of decay and their either opportunistic/saprophytic or parasitic strategy to infect different host tissues. Therefore, different *Armillaria* species have different abilities to colonize a tree, resulting in different severities of fungal infection concerning lesion characteristics and anatomical changes in phloem and cambial tissues. Some virulent species, *A. ostoyae* and *A. mellea*, can even colonize the sapwood and the heartwood of a tree discriminately [177,178]. The infected tree shows a decline in vigour, little shoots, dwarfed leaves and sudden change of leaf colour in autumn. Infected plants generally die some years after infection [179,180]. Efficient colonization of the root collar and the entire circumference may also lead to sudden dieback of the host.

When monitoring forest trees for signs of an armillarioid infection, the appearance of the characteristic mycelial fans under the bark of the basal trunk is a reliable indication that the fungal mycelium is already present in the root system [181]. Then the fungus can also expand into the inner bark of both the roots and trunk, and subsequently cause root lesions and basal canker at the base of the trunk, known as root rot, collar rot or foot rot [25]. Basal resinosis at the root collar and dead cambium are also dependable symptoms indicating *A. ostoyae* and *A. solidipes* infection of various resinous tree species [182,183,184].

Aboveground symptoms that are suggestive of an already impacted root and vascular system due to *Armillaria* infection are wilting, chlorosis, dwarfed or downward-hanging foliage, leaf abscission resulting in premature defoliation, dwarfed fruit, resinosis, little shoots, stand-structural changes, lower-stem deformations, down-wood accumulations, crown thinning and branch dieback, and trees usually die prematurely in the case of conifers as well as nut and fruit crops [17,22,185]. As the trees die, the *Armillaria* inoculum incubates further in their decaying root systems, then it spreads and kills other adjacent susceptible hosts that may lead to massive-scale mortality and the formation of the canopy gaps [186]. Gaps associated with root disease can enlarge, triggered by coalescence of multiple smaller gaps. Among all the factors predicted to directly affect canopy gap size in a pristine ponderosa pine (*Pinus ponderosa*) stand in the Black Hills of South Dakota, *Armillaria* root disease seemed to have the most considerable overall impact, followed by other small-scale disturbances (bark beetles, weak pathogens, ice/snow damage, lightning and wildfires) [185]. Disease centres caused by *A. solidipes* were observed and investigated in West-Central Alberta, Canada, where dead lodgepole pine (*Pinus contorta*) trees appeared commonly in the central infected areas [186]. Diseased trees infected with *Armillaria* are generally smaller than healthy trees for all measured variables, in respect of diameter, height, sapwood area at the base of the live crown, crown width and length [15]. As compared to uninfected trees, symptomatic trees were tested to experience a sustained 5 to 15 years decline in the basal area before death in upland black spruce (*Picea mariana*) forests [187].

### 5.3. Susceptibility of the Host and Plant Defense Mechanisms Associated with Armillaria Infection

Recently, genome-level evolutionary studies confirmed that *Armillaria* and *Desarmillaria* species have evolved from saprotrophic white-rot ancestors towards facultative parasitism [73]; possibly when predecessors of pathogenic species, being well-adapted to wood decay conditions, gained access to the nutrient-rich living tissues of their hosts. Current armillarioid species exhibit a full spectrum of plant–fungus interaction lifestyles ranging from long-term saprotrophic survival under oxygen-limited soil environments through rare symbiotic interactions with mutualist orchid partners [1,163] to aggressively invading and killing vigorous, young trees. Various isolates of parasitic species may exhibit different virulent or non-virulent abilities, and their invasive efficiencies are also dependent on the susceptibility and vigour of their hosts [107,188,189].

The concept of assessing plant fitness towards pathogens is based on a two-component defence response model involving resistance and tolerance. Plants, including forest trees, initially rely on constitutive structural and biochemical defences [190], where the outcomes of the host–pathogen interactions may well be influenced by host-specific and environmental factors [191]. Host–pathogen tests demonstrating tissue-level interactions and degree of pathogenicity have been reported for several *Armillaria* species.

Periderm and rhytidome tissues of stem and root bark play an essential role in the protection against *Armillaria* invasion. When the advancing fungal mycelia reach phellogen in the healthy bark, it induces a series of anatomical changes to limit the growth of the pathogen and replace infected meristems and other tissues. The activated defence response leads to the development of lignified impervious tissue (IT), necrophylactic periderm formation (NP) and callus tissue generation, which is then involved in the compartmentalization of infected tissue and formation of new vascular cambium inside the host plant [175,192]. Different patterns in cambial damage and xylem compartmentalization reflected the susceptibility levels of plant species towards the *Armillaria* pathogens [175]. At similar levels of inoculations, both *A. solidipes* and *A. sinapina* were found equally pathogenic on Douglas-fir, western hemlock and western redcedar; however, based on using inoculum blocks with fungal exudates, *A. solidipes* mycelia were advancing more virulently than those of *A. sinapina* [102]. Equal levels of inoculation, using vegetative fungal mycelia, indicated that both species have a comparable pathogenic potential in contacting and invading their hosts. In contrast, inoculum blocks with fungal exudates, possibly exposing all secreted protein-based and metabolic factors, enforced the real virulent abilities of *A. solidipes* towards host tissues. *A. solidipes* inoculations with exudates appeared to cause lesions on the roots, IT and NP developed in the bark at higher frequencies than *A. sinapina* inoculations, and large proportions of the roots showed no signs of host response. Furthermore, as an indication of possible host-specific communication, *A. solidipes* induced more intense host responses in western redcedar than those following infections by *A. sinapina* [102].

Pathogenicity tests for *A. ostoyae*, *A. mellea* and *A. gallica* on different oak trees were conducted by Sicoli et al. [20], the results indicated that *A. mellea* and *A. gallica* were significantly more virulent on seedlings and young trees of 5 tested *Quercus* species than *A. ostoyae*. One more vital clue came from plant polyphenols, the secondary metabolites acting as the primary chemical defence to inhibit the parasitic fungal growth by restricting the production of cell-wall-degrading enzymes. Hydrolyzable tannins as one type of plant polyphenols are most abundant in the wood, bark, and leaves of *Quercus* species. However, in contrast to *A. ostoyae*, *A. gallica* was shown to be more efficient in oxidizing and metabolizing polyphenols [193].

The age of the tree may also affect plant defence responses. Old and young trees are at a higher risk to be infected by *Armillaria*: in a population of maritime pine infected by *Armillaria*, trees between 10 and 20 years of age displayed fewer symptoms than those below 10 or more than 20 years [168].

## 6. Biotic Factors Facilitating *Armillaria* Transmission and Infection

The establishment and severity of Armillaria root rot disease depend on many interacting factors. Abiotic factors include climatic influences like rainfall, wind speed, sun exposure, and especially the perspective of changing climate and extreme weather. Climate changes, such as the occurrence of flooding, drought, storms or warming, are significant causes of poor soil condition and tree stress, while abiotic soil factors, including oxygen, water, organic and mineral content, pH and temperature, play a vital role in the health of the plant, particularly the root system, which has been discussed in detail elsewhere [1,194,195]. Climate change and poor soil either weaken plant vigour, rendering the plants sensitive to the infection of armillarioid fungi, or directly influence the survival, development, reproduction, and distribution of the pathogens, as well as indirectly changing the abundance of stimulators and competitors of armillarioids in forests [196,197,198]. In this review we focus on biotic factors (fungi, insects and forest conditions) supporting the spread and pathogenicity of armillarioids.

### 6.1. Fungi Stimulating Armillaria Infection

A series of primary pathogenic fungi stimulate armillarioid species to infect trees as secondary pathogens, which has been extensively reviewed by Wargo and Harrington [194]. Fungal diseases with causal agents predisposing their hosts to armillarioid infection include butt rot caused by *Phaeolus schweinitzii* in Douglas-fir, black stain root disease by *Ophiostoma wageneri* (syn. *Leptographium wageneri*) in conifers, sapstreak disease by *Davidsoniella virescens* (syn. *Ceratocystis virescens*) in sugar maple, defoliation by the powdery mildew *Erysiphe alphitoides* (syn. *Microsphaera quercina*) in English oak (*Quercus robur*), beech bark disease by *Neonectria faginata* (syn. *Nectria coccinea* var. *faginata*) in beech, blister rust by *Cronartium ribicola* in western white pine [194], and dieback by the invasive pathogen *Hymenoscyphus fraxineus* in ash (*Fraxinus* spp.) [13,199]. Armillarioids also frequently co-occur with *Heterobasidon annosum*, and the parasitic plant dwarf mistletoe (*Arceuthobium* spp.) predisposes conifers to both of these pathogens [194].

The fungal communities of the tree rhizosphere, stumps, roots, and some woody debris may also include stimulants of armillarioids, which can therefore be considered as essential risk factors of armillarioid invasion [200,201,202,203,204]. Their presence in coniferous and deciduous wood may contribute to the spread of armillarioids and their colonization on stumps. These fungi include *Aspergillus kanagawaensis*, *Aureobasidium pullulans*, *Cylindrocarpon* species, *Chrysosporium* species, *Hormiactis candida*, *Mortierella* species, *Monodictys lepraria*, *Bionectria grammicospora* (syn. *Nectria grammicospora*), *Pseudogymnoascus roseus*, *Penicillium* species, *Phialophora cyclaminis*, *Sporothrix schenckii*, *Pleotrichocladium opacum* (syn. *Trichocladium opacum*) and *Mucor moelleri* (syn. *Zygorhynchus moelleri*) [200,201,202,203,204]. The stimulants are common fungi which occur worldwide, particularly in temperate zones, and possess the ability to stimulate the formation and growth of *Armillaria* rhizomorphs [201]. They increase the weight and length of rhizomorphs and the number of rhizomorph apices. An investigation regarding the stimulatory effects has confirmed that metabolites present in stimulants play an essential role in the growth of *A. ostoyae*: Tryptophol, an indole-3-ethanol analogue, produced by *Mucor moelleri*, acted as a growth-promoting substance and stimulated rhizomorph growth [201]. Kubiak et al. [195] hypothesized that rhizomorphs may be colonized by endogenous fungi and bacteria stimulating hyphal growth of armillarioids and aiding host cell wall degradation by the secretion of extracellular enzymes, which, however, still needs confirmation. It can also be hypothesized that stimulation may also be achieved by microorganisms indirectly, by the inhibition of the natural biological control agents of *Armillaria*.

### 6.2. Interactions between Armillarioids and Insects

Insect pests that are highly destructive in Northern Hemisphere forests, like gypsy moth (*Lymantria dispar*), maple webworm (*Tetralopha asperatella*), eastern and western spruce budworm (*Choristoneura fumiferana* and *Choristoneura occidentalis*, respectively), oak leaf tier (*Acleris semipurpurana*), linden looper (*Erranis tiliaria*), larch casebearer (*Coleophora laricella*), European spruce needleminer (*Epinotia nanaxa*), saddled prominent caterpillar (*Heterocampa guttavitta*), Warren’s rootcollar weevil (*Hylobius warreni*), balsam woolly adelgid (*Adelges piceae*), twolined chestnut borer (*Agrilus bilineatus*), mountain pine beetle (*Dendroctonus ponderosae*), western balsam bark beetle (*Dryocoetes confusus*), fir engraver (*Scolytus ventralis*), spruce wood engraver (*Pityogenes chalcographus*), eight-toothed European spruce bark beetle (*Ips typographus*) or double-spined bark beetle (*Ips duplicatus*) may also be associated with armillarioid root rot ([183,194,195,205,206,207,208,209,210,211,212,213,214,215,216,217,218,219,220,221,222,223,224,225,226,227,228,229,230,231,232,233,234,235], Table 1). Most of the reports about *Armillaria*-insect co-occurrence presumed that defoliating insects (e.g., gypsy moth, maple webworm, eastern spruce budworm, saddled prominent caterpillar) predispose their hosts to *Armillaria* infection, and suggest that defoliating insect damage weakens the trees and increases their susceptibility to armillarioid root rot. This was experimentally supported by Wargo and Houston [235], who inoculated sugar maple trees, defoliated artificially, or naturally by larvae of the saddled prominent caterpillar, with an isolate of *A. gallica*, and found that successful invasion of the root systems depended on stress from defoliation. In the case of root collar weevils (e.g., *Hylobius warreni*, *H. pinicola*) it was proposed that feeding wounds made by these weevils may be important infection courts for armillarioids [227]. A different successional relationship of the co-occurring insect pest and armillarioid root rot pathogen is characteristic for another group of insects including bark beetles (e.g., eight-toothed European spruce bark beetle, fir engraver, western balsam bark beetle), where the armillarioid infection precedes, and also seems to predispose insect damage (Table 1). The observations from lodgepole pine stands growing in Wasatch National Forest of Utah, in the Western USA revealed that many trees attacked by endemic mountain pine beetle (*Dendroctonus ponderosae*) population had roots with *A. mellea sensu lato* infection [229]. Kulhavy et al. [228] postulated a hypothetical sequence of western white pine invasion by blister rust caused by *Cronartium ribicola*, followed by infection of *Armillaria* root rot, and finally the attack of the bark beetles *D. ponderosae* and *P. fossifrons*. Sierota and Grodzki [236] proposed a hypothetical fungal survival strategy for *Armillaria*: Norway spruce trees are first stressed by soil drought and the disappearance of mycorrhizas, making them susceptible to necrotrophic *Armillaria* attack, which results in the release of volatile compounds from resin and phloem attracting engraver beetles (*I. typographus*). The subsequent beetle invasion kills the tree and provides a substrate source for the saprotrophic stage of *Armillaria* [236]. The results of an early study by Madziara-Borusiewicz and Strzelecka [217] seem to back up this hypothesis: The authors found that increased volatile oil amounts of changed chemical composition were produced in spruce needles during the initial phase of *Armillaria* colonization, which was then followed by *Ips* invasion. Among the detected volatile oils, myrtenol is known as one of the main components of attractants and aggregation pheromones of certain bark beetles, suggesting that attraction of engraver beetles by the tree may be connected with the production of host volatiles affected by *Armillaria* [217].

### 6.3. The Role of Forest Condition in Armillarioid Infection

The conditions of a forest, such as biological diversity, host tree density, pathogen inoculum potential, stand history, resistance and resilience to disturbances and sustainable productivity, are also important factors determining the extent to which *Armillaria* pathogens can colonize a forested landscape. Forests with high plant species diversity are more tolerant to *Armillaria* infection, which is even more important as they limit the colonization and spread of pathogens [9]. The virgin forests are rather occupied by the saprotrophic *Armillaria* species, such as *A. gallica* and *A. cepistipes* [108]. Once *A. mellea* colonized the young trees at the edge of a ponderosa pine park, new plantation areas with uniform tree species, the fungus spread rapidly possibly through root contacts, resulting in further infection centre enlargement [103].

Tree species composition, maturity and provenance also play essential roles in the widespread distribution of *Armillaria* pathogens, that are common in the soil of forest area, and no woody plant has complete immunity to infection. However, it has been strongly suggested that seedlings from natural regeneration are less susceptible to *Armillaria* infection than the planted seedlings [237]. The rate of *Armillaria*-induced decay was generally lower in angiosperm than gymnosperm wood types [238]. Conifers with rapid early growth after planting had the most significant mortality closely associated with how quickly primary inoculum of *A. solidipes* transferred to surrounding trees [10]. Conifer seedlings of interior Douglas-fir originated from biologically and physically different environments were screened by Cruickshank et al. [239]. Seedlings originated from warmer and drier places showed lower susceptibility to *A. solidipes* infection, reflected from the ability to limit the spread of the pathogens in the root system. Both tolerance and resistance of the interior Douglas-fir were detected on the seedlings challenged with *A. solidipes*. Tolerant juvenile trees displayed better growth compared with the resistant group, whereas resistant juvenile trees showed less root collar girdling [240]. Investigation of the difference in susceptibility between oak species to *A. mellea* and *A. gallica* revealed that holm oak (*Quercus ilex*) seedlings were most susceptible and Turkey oak (*Q. cerris*), Macedonian oak (*Q. trojana*) and English oak were the least susceptible [241].

## 7. Towards the Biological Control of Armillarioid Root Disease

Since *Armillaria* can persist on infected forest sites for millennia, eradication is practically futile [155]. What, if anything, could or should be done with the productive growing sites for various coniferous or broad-leaved species that are progressively dying from root rot disease caused by armillarioid pathogens? The disease remains inconspicuous until plants with visible and distinct symptoms are observed, and during the period between the infection and appearance of visible symptoms, *Armillaria* may widely spread both in the soil and the host plant [242]. The control of *Armillaria* root disease is also challenging due to the hidden growth of the pathogen in the soil and its persistence in dead plant tissues for decades [22]. The mycelium, beneath the plant bark or inside dead wood, gets protected from the action of control agents [243,244,245].

Among silvicultural practices, planting resistant tree species, inoculum removal realized by removing diseased trees and uprooting even neighbouring uninfected stumps, root collar excavation and solarization may be effective ways of controlling *Armillaria* infections, but do not seem to guarantee a long-term controlling effect and warrant their cost [22,246,247,248,249,250,251,252,253,254,255,256]. Chemical soil fumigants, such as carbon disulfide, methyl bromide, metham-sodium or chloropicrin, as well as non-phytotoxic fungicides seem to be able to control armillarioid infections even in susceptible plants, however, their application is very costly and labour intensive, and it also faces a lot of safety and health issues for the workers and farmers [39,257]. Furthermore, as most of the currently available chemical means for controlling armillarioids are either ineffective or banned, there is an emerging need for effective biocontrol strategies applied either alone or in combination with other control measures [190,258].

An understanding of interactions between plant pathogens and antagonistic organisms in natural environments is crucial for the identification of potential biocontrol agents (BCAs). The application of naturally occurring bacteria, fungal antagonists, as well as nematodes or plant-derived substances may have substantial potential for successfully reducing the pathogenic activities of *Armillaria* [22].

### 7.1. Bacteria

Bacteria have proven effective against several fungal pathogens of agronomic crops and forest trees [259], but in significantly fewer cases against armillarioid forest pathogens. Bacterial antagonism is achieved by different mechanisms, including antibiosis, competition for nutrients and resistance induced in the host [260]. Other factors that influence the efficacy of biocontrol bacteria are their capacity to colonize the rhizosphere or the host and to adapt to soil conditions.

The potential antagonistic behaviour of fluorescent pseudomonads isolated from soils of birch and Douglas-fir stands, and their potential linkage with tree species susceptible to *Armillaria* was investigated by DeLong et al. [261]. It was found that paper birch provides a more favourable environment for these bacteria than Douglas-fir, and fluorescent pseudomonads positively influence the susceptibility of the managed forest stands to *Armillaria* root disease. Several isolates of fluorescent bacteria significantly reduced the growth of *A. solidipes* in paper birch, Douglas-fir, and paper birch—Douglas-fir mixtures [261]. *Pseudomonas* spp. along with *Bacillus*, *Enterobacter*, *Serratia* spp. and *Rhizobium radiobacter* (formerly *Agrobacterium radiobacter*) were isolated from root-free soils of the boreal mixed wood forest of Ontario and found to be capable of inhibiting the linear growth of *A. solidipes* in vitro [262]. Only a few *P. fluorescens* and *Bacillus* isolates were able to prevent in vitro rhizomorph formation of *A. gallica*, indicating their lower ability to suppress the spread of *Armillaria* spp. which produce rhizomorphs more consistently. Gram-negative bacteria, especially members of the genus *Pseudomonas* were observed in decaying fruiting bodies of *A. mellea* and *Coprinus comatus* during the period of their maximum development in forest biocenosis. Such bacterial communities may be promising for the targeted search for bacteria with biocontrol potential [263]. Native biocontrol bacteria (*Pseudomonas fluorescens*, *Bacillus simplex* and two strains of *Erwinia billingiae*) were selected based on their high level of antagonism against the pathogens *A. mellea* and *H. annosum* in Monterey pine seedlings in vitro [264]. These rhizobacterial strains reduced the pathogenic effects of *A. mellea* and the presence of *H. annosum*, exerting antibiotic effects on the fungi. Five isolates of rhizospheric actinobacteria belonging to the species *Streptomyces aurantiacogriseus*, *S. setonensis*, *S. kasugaensis* and *S. jumonjinensis* (two isolates), inhibited the rhizomorph production of *Armillaria* [265]. Actinobacteria also show mutualistic interactions with mycorrhizal fungi along with their antagonism against fungal root pathogens [266].

Soil inoculants produced by a compost fermentation process contain viable populations of bacteria that may serve as antagonists of *A. mellea.* A commercial soil inoculant, Vesta (Biologically Integrated Organics, Inc., Sonoma, CA, USA), was tested against *A. mellea* infection in grapevines and found to be inhibitory under in vitro condition, while at the same time it failed to control Armillaria root disease in vivo but could provide a therapeutic benefit by improving the productivity of infected plants [179].

### 7.2. Fungi

*Trichoderma* species are well known for their antagonistic activity against several fungal plant pathogens [267,268]. Their antagonistic behaviour may be the result of competition [269], antibiosis and direct mycoparasitism [270]. The main challenge in using *Trichoderma* species as BCAs is to maintain an adequate population after the first inoculation, which is required to manage *Armillaria* [251]. The population of *Trichoderma atroviride* strain SC1 was found to decrease over time and eventually reached levels comparable to the natural presence of *Trichoderma* species [271]. Introducing BCAs and promoting their establishment through bark mulch carrier could represent an alternative or complementary strategy for the control of Armillaria root disease, as bark mulch might be useful in maintaining the viability of the introduced *Trichoderma* spp. in the soil for a long time [272].

Microphotographs revealed by scanning electron microscopy indicated the penetration of *Trichoderma* hyphal tips into the melanized outer tissue of the rhizomorphs of host fungi, and *Trichoderma* eventually killed the *Sclerotium rolfsii*, *Rhizoctonia solani* and *Armillaria gallica* hyphae by coiling and lysis of hyphal cells [273,274]. The metabolites produced by *Trichoderma* spp. exhibited toxicity to the causal agent of Armillaria root of tea (*Camellia sinensis*) in Kenya [275]. Furthermore, *Trichoderma* spp. excrete mycolytic enzymes for the digestion of the cell wall of the target fungus, which causes the leakage of the cytoplasm from the host cells resulting in their lysis. The host cytoplasm is, apparently, utilized by the mycoparasite for its further spread [273].

The strong antagonism of *T. citrinoviride* to *A. solidipes* seemed to be induced by diffusible compounds that inhibited the growth of *A. solidipes* and the formation of rhizomorphs. It was also proved that these compounds were metabolites and not enzymes since they could still suppress the growth of *A. solidipes* after denaturing any enzymes in the filtrates of *Trichoderma* isolates during autoclaving [276]. A further study indicated that compounds from the fermentation of *T. longibrachiatum* and *T. harzianum* in different media exhibited antibacterial and antifungal activities [277]. 6-*n*-pentyl-α-pyrone (6-PP) was the most active metabolite, at a concentration of 200 ppm completely suppressing the growth of *A. mellea*. Sorbicillin showed moderate antifungal activity on the fungi *Paecilomyces variotii* and *Penicillium notatum* but no activity against *A. mellea* [277].

The biocontrol interactions were studied by detecting metabolic assimilation of *T. atroviride* SC1 from a ^13^C-labelled *A. mellea* using isotope ratio mass spectrometry (IRMS) in dual-culture tests. The results showed that, during the direct contact with ^13^C labelled *A. mellea*, the ^13^C content in the mycelia of *T. atroviride* increased significantly by assimilating some leaching exudates and metabolites of the pathogen, but mostly assimilating from actively parasitizing the pathogen [270]. A similar study using the same method was conducted for *Trichoderma harzianum*, which inhibited *A. mellea* with a growth rate of inhibition 80 ± 0.19%. During contacting with ^13^C-labelled *A. mellea*, ^13^C values of *T. harzianum* reached to a significantly higher level than the assimilation of ^13^C in the antagonistic bacteria *Rhodosporidium babjevae* and *Pseudomonas fluorescens*. The mycoparasitic activity of *T. harzianum* against the labelled pathogen sustained for one month in dual culture [278].

In a glasshouse experiment, the isolate Tham1 of *Trichoderma hamatum*, the isolate Th23 of *T. harzianum* and the *T. viride* isolate Tv3, grown on either sterile wheat bran or mushroom compost, showed a protective effect on the potted strawberry plants against *A. mellea*. Application of the *Trichoderma* antagonists resulted in healthier plants which developed significantly more leaves [279].

The isolate SC1 of *Trichoderma atroviride*, as an experimental biocontrol agent, provided effective control of vinegrape root rot disease caused by *A. gallica* and *A. mellea* [280]. *T. atroviride* SC1 grew sustainably on the barks of different plant species, such as larch, fir and pine for an extended period, up to 16 weeks. The best survival rate of this antagonist was detected on the bark mixture of these species. Bark pre-inoculated with *T. atroviride* SC1 was applied as mulch to strawberry; as a result, it significantly reduced the extent of root colonization by *A. gallica* on strawberry plants [88]. Application of *T. harzianum* to the soil surrounding the wood-borne inoculum of *Armillaria* caused a significant reduction in the viability of the pathogen [255]. *Armillaria* failed to invade the stem sections colonized by *T. harzianum* and had low viability in the plant materials inoculated with *Trichoderma* [256]. The use of air-spading combined with *T. harzianum* inoculation also proved to be a potential joint cultural/biocontrol strategy against *A. mellea* in a forest [281]. Chen et al. [282] performed a large-scale screening approach to identify potential biocontrol candidates among *Trichoderma* strains isolated from healthy and *Armillaria*-damaged forests. The isolates were examined for in vitro antagonistic abilities towards *Armillaria* species as well as for the production of siderophores and indole-3-acetic acid, resulting in the selection of a *T. virens* and *T. atrobrunneum* strain, which were tested under field conditions where their application revealed better survival of Turkey oak seedlings under *Armillaria*-infested soil conditions. Rees et al. [283] focused on the isolation of endophytic *Trichoderma* strains and their investigation regarding the potential to control *A. mellea*, and found that strains of *T. virens* and *T. hamatum* possessed the best antagonistic abilities on pre-colonized hazel disks.

Although BCAs are considered to be safer than chemicals, large populations of a microbial BCA may also have adverse effects on non-target species of the microbiome [284]. A persistent and aggressive BCA may pose risks to the natural microbiota, as it is more competitive for nutrients than many other soil microorganisms [285]. The application of *T. atroviride* SC1 also posed a low risk to non-target bacterial and fungal populations in soil microbiota of a vineyard in Italy [280].

The rapidly growing saprophytic basidiomycete fungi have also proven effective as biocontrol agents to reduce or even prevent armillarioid invasion and colonization by decreasing the available stump wood base through aggressive spatial competition [286]. Mycelial cord-forming fungi have shown a considerable potential to colonize woody debris under field conditions and persist in woodland forests for a long time [287,288]. Competitive antagonists, viz. *Hypholoma fasciculare*, *Schizophyllum commune*, *Ganoderma lucidum*, *Xylaria hypoxylon* or *Phanerochaete velutina* can overgrow the colonies of *D. tabescens* and *A. mellea*, ultimately reducing the inocula, thus decreasing the threat to adjacent trees and/or subsequent plantings [123]. Promising competitors may even be found within the genus *Armillaria* itself: *A. altimontana* was found to be harmless to western white pine but reported to frequently co-occur with the virulent primary pathogen *A. solidipes* in Northern Idaho, suggesting that non-harmful *Armillaria* species may have in situ biocontrol potential against their root rot pathogenic relatives [81].

Mycorrhizal associations have been found helpful in enhancing the resistance of plants to certain fungal pathogens [289,290,291,292,293]. In these symbiotic relationships, the fungus receives carbohydrates from its host, while the plant gets benefited from multiple positive effects of the association. Earlier it has also been reported that ectomycorrhizal fungi can reduce the infections caused by *Armillaria* [294]. In vitro interaction studies between arbuscular mycorrhizal fungi (AMF) and *A. mellea* were conducted in grapevine [295]. As a result, AMF symbiosis was found to improve the tolerance of grapevine to *A. mellea* without showing any direct antagonism or antibiosis against the pathogen, which suggested that the defensive response of grapevines against *A. mellea* must be indirect, mediated through the host plant physiology [295].

### 7.3. Nematodes

Certain nematodes were also found to adversely affect the growth of *A. mellea* infecting ponderosa pine seedlings [296] and used as alternative biological agents against *Armillaria* [297]. *Aphelenchus avenae*, a mycophagous nematode, reduced and eventually stopped the growth of *A. mellea* in vitro [298]. Further research is needed for the identification of suitable mycophagous nematodes against *Armillari*a species, as the population of *Aphelenchoides*, *Aphelenchus*, *Ditylenchus* and *Neotylenchus* spp. is relatively high in several crops [299,300].

### 7.4. Substances Derived from Cyanobacteria and Plants

Cyanobacteria are essential sources of novel antifungal compounds [301,302]; the methanolic extract of a *Nostoc* strain showed an inhibitory effect to *Armillaria* spp. [303]. *Nostoc* strain GSV224 was found to be an excellent cryptophycin-producer during the screening of cyanobacterial anticancer activity [304]. Cryptophycin-1 has the ability to bind to the ends of the microtubules, suppressing the microtubule dynamics, thereby blocking the cell cycle at the metaphase of mitosis [305]. The lack of literature on its use in agriculture indicates the gaps in knowledge about the possible role of cryptophycin in limiting the spread of *Armillaria*.

Several allelopathic compounds were found to be effective against plant pathogens and herbivores, and they can also influence soil microorganisms [306]. Some invasive plants can change the soil microbial communities, which can improve the growth of the invasive species or harm the native microbiota [307,308]. Antifungal activity of aqueous and organic extracts from different plants and their products against *Armillaria* spp. are being tested, but no significant inhibitory effect was observed so far. This idea needs further and specialized research in the future to identify BCAs effective against *Armillaria* infections. The antifungal activities of organic extracts derived from invasive and indigenous goldenrod (*Solidago* spp.) proved to be weak against Armillaria root disease [309].

Biofumigation can be carried out by incorporation of broccoli (*Brassica oleracea* var. *italica*) residues into the soil. The antifungal properties of biofumigants have been associated with their high glucosinolate content, which can be hydrolyzed to release antifungal isothiocyanates [310]. Isothiocyanates released from the hydrolysis of the glucosinolate sinigrin, isolated from *Brassica* seed, showed antifungal properties against *A. mellea* strains in in vitro experiments [311]. Sinigrin has shown a fungicidal effect at a concentration of 100 μM, while lower concentrations resulted in a temporary fungistatic effect. Moreover, the application of biofumigant formulations obtained from *Brassica* seeds on potted peach plants inoculated with *A. mellea* resulted in enhanced soil biological activity (basal respiration, soil nitrate concentration and microbial biomass) as well as increased peach plant growth, chlorophyll concentration and leaf nitrogen [311]. However, the inhibition of *A. mellea* growth by the application of *Brassica* seed meal in in vivo trials was not evidenced due to the lack of infection symptoms in experimentally inoculated potted trees, thus further research is required to study its efficacy in Armillaria root rot disease control. Allicin (diallyl thiosulfinate), a stabilized garlic extract product, was tested on 100 isolates of *A. gallica* and *A. mellea* and was found to inhibit the growth of both fungi under in vitro conditions [312]. The potential of allicin for field use is limited due to better inhibition of the less virulent *A. gallica* than the more aggressive *A. mellea.*

### 7.5. Combined Approaches

Ample research has been performed on the feasibility of integrated control of *Armillaria* spp. Wise and judicious use of fire followed by the inoculation of *T. harzianum* and *T. citrinoviride* reduced the growth and rhizomorph formation of *A. solidipes* and might be used as an option for the integrated strategy of managing armillarioid root diseases [276]. It has also been proved earlier that fire enhanced the concentration of cations in the soil which come from the ash layer, negatively affecting the growth and development of *A. solidipes* in vitro [313,314]. If the soil is warmer and drier, there is a lower probability of survival of *Armillaria* mycelium within partially decayed roots, encouraging the mycoparasitism on *Armillaria* mycelium by soil-borne fungi like *Trichoderma* species [315].

Rhizomorphs of *Armillaria* spp. play a pivotal role in infecting healthy plants and ultimately spread the disease. Root collar excavation followed by *Trichoderma* inoculation appears to offer a promising integrated strategy for the management of *A. mellea* [18,281,315]. The method alone is unlikely to eradicate an *Armillaria* infection, but in the case of grapevine, it may allow an infected plant to tolerate Armillaria root rot [315].

The non-volatile metabolites of *T. harzianum* in combination with sulphate salts of Al^2+^ and Fe^3+^ reduced the number of rhizomorphs of *A. borealis* and *A. gallica*, respectively [316] and thus may be tested for a future integrated strategy.

The application of soil-borne *Trichoderma* spp. may be more effective in controlling *Armillaria* infections after the fumigation of soil with methyl bromide and carbon disulfide, as sublethal doses of these fumigants seem to make the mycelium more vulnerable to mycoparasitism by *Trichoderma* spp. [317,318]. Moreover, *Trichoderma* strains can survive at much higher concentrations of soil fumigants than *A. mellea* [22,39,317,319,320], the population of *Trichoderma* spp. presumably seemed even increased by field fumigation with methyl bromide [319,321]. Moreover, when the root pieces fumigated with carbon disulphide were buried in unsterilized soil or soil amended with *Trichoderma*, *A. mellea* was killed and replaced more effectively [322].

The integration of several isolates of *T. harzianum* with two systemic fungicides, Fosetyl-Al (Aliette) and Fenpropidin, was tested to suppress the *Armillaria* root disease in potted strawberry plants in a greenhouse experiment, and a significant interaction was observed among the *Trichoderma* spp., fungicides and the timing of their application [318]. *T. harzianum* isolates Th2 and Th23 were generally more effective against *Armillaria* when applied with a time interval of 40 days after Fenpropidin or before Fosetyl-Al. Moreover, Fosetyl-Al was found to be significantly more effective than Fenpropidin in enhancing the survival of strawberry plants even when used in high amounts, while Fenpropidin in its high doses was observed to be phytotoxic to strawberry plants [318]. The active ingredient of Fosetyl-Al is phosphonic acid, which has already been proven as a control agent against *Armillaria* [39].

## 8. Conclusions

The economic and environmental problems caused by *Armillaria* species worldwide, especially in the Northern Hemisphere, increase the need for efficient solution strategies, the development of which requires a solid genome-level knowledge about the molecular background of the pathogenic activities of various species and the infection process of their virulent representatives, as well as the composition and function of the microbiota associated with them in their natural environments. In the “omics” era, modern tools of molecular biology enabling the realization of the above goals are becoming widely available. Genome-level analysis of orthologues offers the possibility of resolving the taxonomy for armillarioids at genus, clade, and species levels. Beyond that, high-quality genomes and well-controlled plant–fungus interaction tests are in line to develop genome-based pathogenicity models, while metatranscriptomic analyses of native infestation assays may assist further in identifying the microbial components contributing to or controlling the invasive activities of various *Armillaria* mycelia.

## Figures and Tables

**Table 1:** Insect pests co-occurring with armillarioid root rot in the Northern Hemisphere.

Co-occurring Insect Pest	Armillarioid Species	Location	Host Tree	Year	Reference
**Lepidoptera**					
Gypsy moth (*Lymantria dispar*)	*Armillaria* sp.	Massachusetts, USA	Oak	1912–1921	[205]
	*Armillaria* sp.	New Jersey, USA	Oak	1967	[206]
	*Armillaria* sp.	Pennsylvania, USA	Oak	1985	[207]
	*Armillaria* sp.	Maryland, USA	Oak	1985–1987	[208]
	*A. gallica*	Pennsylvania, West Virginia, Maryland, USA	Oak	1993	[209]
Eastern spruce budworm (*Choristoneura fumiferana*)	*A. mellea sensu lato*	Canada	Balsam fir	1955–1958	[210]
	*Armillaria* spp.	New Brunswick, Canada	Balsam fir	1960s	[211]
	*Armillaria* spp.	Newfoundland, Canada	Black spruce	early 1980s	[212]
Western spruce budworm (*Choristoneura occidentalis*)	*A. altimontana* (NABS X)	Eastern Oregon, USA	Grand fir	1989	[213]
Maple webworm (*Tetralopha asperatella*)	*A. mellea sensu lato*	Wisconsin, USA	Sugar maple	late 1950s	[214]
Oak leaf tier (*Acleris semipurpurana*)	*Armillaria* sp.	Pennsylvania, USA	Red oak Scarlet oak	1960s	[215]
Saddled prominent caterpillar (*Heterocampa guttavitta*)	*Armillaria* spp.	North-Central New York, USA	Sugar maple	1991	[194]
European spruce needleminer (*Epinotia nanaxa*)	*Armillaria* sp.	Norway	Norway spruce	1984	[216]
Linden loopers (*Erranis tiliaria*)	*Armillaria* sp.	Maryland, USA	Oak	1985–1987	[208]
**Coleoptera**					
Eight-toothed European spruce bark beetle	*A. mellea sensu lato*	South Poland	Spruce	1974	[217]
(*Ips typographus)*	*Armillaria* sp.	Northeastern Slovakia	Spruce	1995	[218]
	*Armillaria* spp.	Western Beskidy, Poland	Norway spruce	2000s	[219]
	*A. ostoyae* *A. cepistipes* *A. borealis*	Bohemian forest, Czech Republic	Norway spruce	2002	[220]
	*A. ostoyae* *A. cepistipes* *A. gallica*	Eastern Czech Republic	Norway spruce	2010s	[183]
Fir engraver (*Scolytus ventralis*)	*A. mellea sensu lato*	Northern Idaho, USA	Grand fir	1972	[221]
	*Armillaria* sp.	Idaho, USA	Grand fir	1974	[222]
	*A. mellea sensu lato*	Colorado, USA	White fir	1981	[223]
	*A. mellea sensu lato*	Oregon and Washington, USA	Grand fir Rocky Mountain white fir Pacific silver fir Noble fir California red fir Subalpine fir	1977	[224]
Western balsam bark beetle (*Dryocoetes confusus*)	*A. mellea sensu lato*	Oregon and Washington, USA	Subalpine fir	1977	[224]
	*A. solidipes*	Central Oregon, USA	Grand fir	1979, 1992	[225]
	*A. mellea sensu lato*	Colorado, USA	Subalpine fir	1981	[223]
Warren’s rootcollar weevil (*Hylobius warreni*)	*A. mellea sensu lato*	Saskatchewan, Canada	White spruce	1960	[226]
	*Armillaria* sp.	Newfoundland, Canada	Sitka spruce Norway spruce Red pine	1970	[227]
Mountain pine beetle (*Dendroctonus ponderosae*)	*A. mellea sensu lato*	Idaho, USA	Western white pine	1975	[228]
	*A. mellea sensu lato*	Utah, USA	Lodgepole pine	1983	[229]
Bark beetle (*Pityogenes fossifrons*)	*A. mellea sensu lato*	Idaho, USA	Western white pine	1975	[230]
Double-spined bark beetle (*Ips duplicatus*) Sixtoothed spruce bark beetle (*Pityogenes chalcographus*)	*A. ostoyae* *A. cepistipes* *A. gallica*	Eastern Czech Republic	Norway spruce	2010s	[183]
Twolined chestnut borer (*Agrilus bilineatus*)	*A. mellea sensu lato*	Connecticut, USA	Oak	1969–1973	[231]
Spruce wood engraver (*Pityogenes chalcographus*) Brown longhorn beetle (*Obrium brunneum*) Spruce shortwing beetle (*Molorchus minor*) *Pogonocherus fasciculatus* *Phthorophloeus spinulosus*	*Armillaria* sp.	Western Carpathian mountain, Czech Republic	Norway spruce	1990s	[232]
**Hemiptera**					
Balsam woolly adelgid (*Adelges piceae*)	*A. mellea sensu lato*	Newfoundland, Canada	Balsam fir	1960s	[233,234]

## Data Availability

Data is contained within the article and Supplementary Materials.

## Supplementary Materials

The following are available online at https://www.mdpi.com/2076-0817/10/1/76/s1, Table S1: Geographical distribution of *Armillaria ostoyae* based on epidemiology data, Table S2: Geographical distribution of *Armillaria solidipes* based on epidemiology data, Table S3: Geographical distribution of *Armillaria sinapina* based on epidemiology data, Table S4. Geographical distribution of *Armillaria gemina* based on epidemiology data, Table S5: Geographical distribution of *Armillaria borealis* based on epidemiology data, Table S6: Geographical distribution of *Armillaria mellea* based on epidemiology data, Table S7: Geographical distribution of *Armillaria gallica* based on epidemiology data, Table S8: Geographical distribution of *Armillaria cepistipes* based on epidemiology data, Table S9: Geographical distribution of *Armillaria calvescens* based on epidemiology data, Table S10: Geographical distribution of *Desarmillaria tabescens* based on epidemiology data.
