# Supplementary material for: Epidemiology, Biotic Interactions and Biological Control of Armillarioids in the Northern Hemisphere
Source: Pathogens. 2021 Jan 16;10(1):76. doi: 10.3390/pathogens10010076 (PMC7830283; doi:10.3390/pathogens10010076)
Supplement: Supplementary file 1 [file pathogens-10-00076-s001.pdf]

Supplementary Table 1. Geographical distribution of *Armillaria ostoyae* based on epidemiology data

Continent	Country of occurrence	Host plant	Year of isolation	Reference
**Asia**	Japan	Field maple (*Acer campestre*), Sycamore (*Acer pseudoplatanus*)<br>Gray alder (*Alnus incana*)<br>Juneberry (*Amelanchier lamarckii*)<br>Birch (*Betula* sp.)<br>Hornbeam (*Carpinus betulus*)<br>Sweet chestnut (*Castanea sativa*)<br>Hazelnut (*Corylus avellana*)<br>Common hawthorn (*Crataegus monogyna*)<br>Beech (*Fagus sylvatica*)<br>Forsythia (*Forsythia* sp.)<br>Alder buckthorn (*Frangula alnus*)<br>Common holly (*Ilex aquifolium*)<br>Austrian pine (*Pinus nigra*)<br>Black cherry (*Prunus serotina*)<br>Common oak (*Quercus robur*), Red oak (*Quercus rubra*),<br>Black locust (*Robinia pseudoacacia*)<br>Rowan (*Sorbus aucuparia*)<br>Linden (*Tilia* sp.)	2005	[1]
		Momi fir (*Abies firma*), Nikko fir (*Abies homolepis*), Sachalin fir (*Abies sachalinensis*)<br>Japanese cypress (*Chamaecyparis obtusa*)<br>Japanese larch (*Larix kaempferi*)<br>Norway spruce (*Picea abies*), Sakhalin spruce (*Picea glehnii*), Dark-bark spruce (*Picea jezoensis*)<br>Japanese red pine (*Pinus densiflora*), Japanese black pine (*Pinus thunbergii*)<br>Northern Japanese hemlock (*Tsuga diversifolia*)	1976-2007	[2]

Supplementary Table 1. - continued

Continent	Country of occurrence	Host plant	Year of isolation	Reference
**Europe**	Albania	Greek fir (*Abies borisi-regis*)<br>Beech (*Fagus sylvatica*)<br>Common juniper (*Juniperus communis*)<br>Norway spruce (*Picea abies*)<br>Austrian pine (*Pinus nigra*), Scots pine (*Pinus sylvestris*)	1990-2006	[3]
	Austria	Spruce (*Picea* sp.)	2016	[4]
	Czech Republic	Norway spruce (*Picea abies*)<br>Beech (*Fagus sylvatica*	unknown	[5]
	England	Norway spruce (*Picea abies)*<br>Corsican pine *(Pinus nigra),* Scots pine (*Pinus sylvestris*)<br>Douglas-fir (*Pseudotsuga menziesii*)	unknown	[6,7]
		Silver birch (*Betula pendula*)<br>Lawson cypress (*Chamaecyparis lawsoniana*)<br>Common ash (*Fraxinus excelsior*)<br>European larch (*Larix decidua*), Japanese larch (*Larix kaempferi*)<br>Norway spruce (*Picea abies*), Purkyne Serbian Spruce (*Picea omorika*), Sitka spruce (*Picea sitchensis*)<br>*Pinus nigra* subsp. *laricio,* Scots pine (*Pinus sylvestris*), Bhutan pine (*Pinus wallichiana*)<br>Douglas-fir (*Pseudotsuga menziesii*)<br>Common oak (*Quercus robur*)<br>Hiba *(Thujopsis dolabrata)*<br>Western hemlock-spruce (*Tsuga heterophylla*)	unknown	[8]

Supplementary Table 1. - continued

Continent	Country of occurrence	Host plant	Year of isolation	Reference
**Europe**	England	Buckeye/Chestnut (*Aesculus* sp.)<br>Birch (*Betula* sp.)<br>Witch hazels *(Hamamelis* sp.*)*<br>Privet (*Ligustrum* sp.)<br>*Prunus* sp.<br>*Rhododendron* sp.<br>Rowan (*Sorbus* sp.)	2004-2007	[9]
		Katsura (*Cercidiphyllum* sp.)<br>Sweetgum (*Liquidambar* sp.)<br>*Prunus* sp.	2004-2007<br>2017	[10]
	France	Silver fir (*Abies alba*), Grand fir (*Abies grandis*)<br>Strawberry tree *(Arbutus unedo)*<br>Common heather *(Calluna vulgaris)*<br>Hazelnut (*Corylus avellana*)<br>Hawthorn *(Crataegus* sp.*)*<br>Provence broom *(Cytisus purgans)*<br>Green heather *(Erica scoparia)*<br>Alder buckthorn (*Frangula alnus*)<br>European larch (*Larix decidua*)<br>Norway spruce (*Picea abies*), Sitka spruce (*Picea sitchensis*)<br>Lodgepole pine (*Pinus controta*), Mountain pine (*Pinus mugo*), *Pinus nigra* subsp. *laricio,* Maritime pine (*Pinus pinaster*), Monterey pine (*Pinus radiata*), Scots pine (*Pinus sylvestris*), Loblolly pine (*Pinus taeda*), Bhutan pine (*Pinus wallichiana*)<br>Douglas-fir (*Pseudotsuga menziesii*)<br>Common oak (*Quercus robur*), Pyrenean oak (*Quercus pyrenaica*)<br>*Rubus* sp.<br>Scotch broom *(Sarothamnus scoparius)*<br>*Gorse (Ulex europaeus)*	unknown	[8]

Supplementary Table 1. - continued

Continent	Country of occurrence	Host plant	Year of isolation	Reference
**Europe**	France	Silver fir (*Abies alba*), Greek Fir (*Abies borisi-regis*)<br>Silver birch (*Betula pendula*)<br>Cypress (*Cupressus sempervirens*)<br>Beech (*Fagus sylvatica*)<br>Common Juniper (*Juniperus communis*)<br>Norway spruce (*Picea abies*), Purkyne Serbian Spruce (*Picea omorika*)<br>Mountain pine (*Pinus mugo*), Austrian pine (*Pinus nigra*), Maritime pine (*Pinus pinaster*), Stone pine (*Pinus pinea*), Scots pine (*Pinus sylvestris*)<br>Wych elm (*Ulmus glabra*)	1990	[11]
		Maritime pine (*Pinus pinaster*)<br>Holm oak (*Quercus ilex*), Common oak (*Quercus robur*)	unknown	[12,13]
	Greece	Silver fir (*Abies alba*)<br>Beech (*Fagus sylvatica*)<br>Norway spruce (*Picea abies*)<br>Scots pine (*Pinus sylvestris*)<br>Douglas-fir (*Pseudotsuga menziesii*)	1991-1996	[14]
	Italy	Silver fir (*Abies alba*)<br>Silver birch (*Betula pendula*)<br>Sweet chestnut (*Castanea sativa*)<br>Lawson cypress (*Chamaecyparis lawsoniana*)<br>European larch (*Larix decidua*)<br>Norway spruce (*Picea abies*)<br>Swiss pine *(Pinus cembra), Pinus nigra* subsp. *laricio,* Maritime pine (*Pinus pinaster*), Scots pine (*Pinus sylvestris*)	unknown	[8]

Supplementary Table 1. - continued

Continent	Country of occurrence	Host plant	Year of isolation	Reference
**Europe**	Italy	Douglas-fir (*Pseudotsuga menziesii*)<br>Common oak (*Quercus robur*)<br>Cedar (*Thuja* sp.)	unknown	[8]
	Netherland	Fir (*Abies* sp.)<br>Beech (*Fagus* sp.)<br>Pine (*Pinus* sp.)	1983-1992	[15]
	Norway	Scots pine (*Pinus sylvestris*)	unknown	[16]
	Poland	European larch (*Larix decidua*)<br>Norway spruce (*Picea abies*)<br>Scots pine (*Pinus sylvestris*)<br>Sessile oak (*Quercus petraea*)	1994	[17,18]
	Poland	Scots pine (*Pinus sylvestris*)	2005-2013	[19]
	Serbia	Silver fir (*Abies alba*)<br>Balkan beech (*Fagus moesiaca*)<br>Norway spruce (*Picea abies*), Purkyne Serbian Spruce (*Picea omorika*)	2002-2007	[20]
	Spain	Austrian pine (*Pinus nigra*), Maritime pine (*Pinus pinaster*), Monterey pine (*Pinus radiata*)	2002-2003	[21]
	Switzerland	Japanese larch (*Larix kaempferi*)<br>Norway spruce (*Picea abies*)<br>Scots pine (*Pinus sylvestris*)<br>Douglas-fir (*Pseudotsuga menziesii*)	1992 and 1999	[22]

Supplementary Table 1. - continued

Continent	Country of occurrence	Host plant	Year of isolation	Reference
**Europe**	Switzerland	Swiss mountain pine (*Pinus mugo* ssp. *uncinata*)	1993-1998	[23]
		Mountain pine (*Pinus mugo*)	2003-2004	[24]
		Scots pine (*Pinus sylvestris*)	unknown	[25]
	Turkey	Silver fir (*Abies alba*)<br>Hornbeam (*Carpinus betulus*), Oriental hornbeam (*Carpinus orientalis*)<br>Balkan Beech (*Fagus moesiaca*)<br>Norway spruce (*Picea abies*), Purkyne Serbian Spruce (*Picea omorika*)<br>Poplar (*Populus* sp.)<br>Hungarian oak (*Quercus frainetto*), Sessile oak (*Quercus petraea*), Turkey oak (*Quercus cerris*)<br>Willow (*Salix* sp.)	2011	[26]
	Ukraine	Beech (*Fagus* sp.)	unknown	[27]

Supplementary Table 2. Geographical distribution of *Armillaria solidipes* based on epidemiology data

Continent	Country of occurrence	Host plant	Year of isolation	Reference
**America**	British Columbia	Subalpine fir (*Abies lasiocarpa*)<br>Douglas maple (*Acer glabrum* var. *douglasii*), Big leaf maple (*Acer macrophyllum*)<br>Red alder (*Alnus rubra*), Green alder (*Alnus sinuata)*<br>Paper birch (*Betula papyrifera*)<br>Hawthorn *(Crataegus* sp.*)*<br>White spruce (*Picea glauca*), Engelmann spruce (*Picea engelmannii*)<br>Lodgepole pine (*Pinus contorta*)*,* Western white pine (*Pinus monticola*)<br>Quaking aspen (*Populus tremuloides*), Black cottonwood *(Populus trichocarpa)*<br>Bitter cherry *(Prunus emarginata)*<br>Douglas-fir (*Pseudotsuga menziesii*)<br>Willow (*Salix* sp.)<br>Western red cedar (*Thuja plicata*)<br>Western hemlock-spruce (*Tsuga heterophylla*)	unknown	[28]*
		Subalpine fir (*Abies lasiocarpa*)<br>Engelmann spruce (*Picea engelmannii*)*,* White spruce (*Picea glauca*)<br>Lodgepole pine (*Pinus contorta, Pinus contorta var. latifolia*)<br>Quaking aspen (*Populus tremuloides*)<br>Douglas-fir (*Pseudotsuga menziesii*)	1997	[29]*
		Western larch (*Larix occidentalis*)<br>Douglas-fir (*Pseudotsuga menziesii* ssp. *glauca*)	unknown	[30]*
		Douglas maple (*Acer glabrum* var. *douglasii*)<br>Sitka alder (*Alnus viridis* ssp. *sinuata*)*,*<br>Paper birch (*Betula papyrifera*)<br>Lodgepole pine (*Pinus controta*)*,* Western white pine (*Pinus monticola*)<br>Quaking aspen (*Populus tremuloides*)	unknown	[31]*

Supplementary Table 2. - continued

Continent	Country of occurrence	Host plant	Year of isolation	Reference
**America**	British Columbia	*Prunus* sp.<br>Douglas-fir (*Pseudotsuga menziesii*)<br>Willow (*Salix* sp.)<br>Western red cedar (*Thuja plicata*)<br>Western hemlock-spruce (*Tsuga heterophylla*)	unknown	[31]*
	Canada	Silver maple (*Acer saccharinum*), Sugar maple (*Acer saccharum*)<br>Golden birch (*Betula alleghaniensis*), Paper birch (*Betula papyrifera*), Grey birch (*Betula populifolia*)<br>White pine (*Pinus strobus*)<br>White oak (*Quercus alba*)	1986	[32]*
		Alder (*Alnus* sp.)<br>Paper birch (*Betula papyrifera*)<br>White spruce (*Picea glauca*), Black spruce (*Picea mariana*)<br>Jack pine (*Pinus banksiana*), Lodgepole pine (*Pinus controta*), Red pine (*Pinus resinosa*)<br>Douglas-fir (*Pseudotsuga menziesii*)<br>Poplar (*Populus* sp.), Balsam poplar (*Populus balsamifera*), Quaking aspen (*Populus tremuloides*)<br>Silver buffaloberry (*Shepherdia argentea*), Canada buffaloberry (*Shepherdia canadensis*)	unknown	[33]*
	Colorado	White fir (*Abies concolor*), Subalpine fir (*Abies bifolia*)<br>Engelmann spruce (*Picea engelmannii*), Blue spruce (*Picea pungens*)<br>Ponderosa pine (*Pinus ponderosa*)<br>Quaking aspen (*Populus tremuloides*)<br>Douglas-fir (*Pseudotsuga menziesii*)	unknown	[34]*
	Massachusetts	Sugar maple (*Acer saccharum*), Red maple (*Acer rubrum*)<br>Golden birch (*Betula alleghaniensis*), Paper birch (*Betula papyrifera*)<br>Pignut hickory (*Carya glabra*)<br>Red oak (*Quercus rubra*), Black oak (*Quercus velutina*)	1988	[35]*

Supplementary Table 2. - continued

Continent	Country of occurrence	Host plant	Year of isolation	Reference
**America**	Massachusetts	Ponderosa pine (*Pinus ponderosa*)<br>Douglas-fir (*Pseudotsuga menziesii*)<br>Gambel oak (*Quercus gambelii*)	unknown	[36]
		Red maple (*Acer rubrum*)<br>Sweet birch (*Betula lenta*), Paper birch (*Betula papyrifera*), Grey birch (*Betula populifolia*)<br>American chestnut (*Castanea dentata*)<br>Pitch pine (*Pinus rigida*), White pine (*Pinus strobus*)<br>White oak (*Quercus alba*), Scarlet oak (*Quercus coccinea*), Common red oak (*Quercus rubra*), Black oak (*Quercus velutina*)<br>Sassafras (*Sassafras albidum*)<br>Eastern hemlock-spruce (*Tsuga canadensis*)	unknown	[37]
	Michigan	Apple (*Malus pumila*)<br>Wild cherry (*Prunus avium*), Sour cherry (*Prunus cerasus*), Peach (*Prunus persica*)	1985	[38]*
		Prunus sp.	2018	[39]*
	Minnesota	Balsam fir (*Abies balsamea*)<br>Red maple (*Acer rubrum*)<br>Paper birch (*Betula papyrifera*)<br>Jack pine (*Pinus banksiana*), Red pine (*Pinus resinosa*)	1991-1992	[40]*
	New Mexico	White fir (*Abies concolor*)<br>Blue spruce (*Picea pungens*)<br>Ponderosa pine (*Pinus ponderosa*), Southwestern white pine (*Pinus strobiformis*)<br>Quaking aspen (*Populus tremuloides*)<br>Douglas-fir (*Pseudotsuga menziesii*)	1988	[41]*

Supplementary Table 2. - continued

Continent	Country of occurrence	Host plant	Year of isolation	Reference
**America**	New York	Balsam fir (*Abies balsamea*)<br>Red maple (*Acer rubrum*), Sugar maple (*Acer saccharum*)<br>Golden birch (*Betula alleghaniensis*), Paper birch (*Betula papyrifera*)<br>American beech (*Fagus grandifolia*)<br>Red spruce *(Picea rubens)*<br>Red pine (*Pinus resinosa*), White pine (*Pinus strobus*), Scots pine (*Pinus sylvestris*)<br>Douglas-fir (*Pseudotsuga menziesii*)<br>Eastern hemlock-spruce (*Tsuga canadensis*)	1986-1989	[42]*
		Balsam fir (*Abies balsamea*)<br>Sugar maple (*Acer saccharum*)<br>Scots pine (*Pinus sylvestris*)<br>Douglas-fir (*Pseudotsuga menziesii*)<br>Black cherry (*Prunus serotina*)	unknown	[43]*
	Ontario	Balsam fir (*Abies balsamea*)<br>Red maple (*Acer rubrum*), Sugar maple (*Acer saccharum*)<br>Yellow birch (*Betula alleghaniensis*), Paper birch (*Betula papyrifera*)<br>Bitternut hickory (*Carya cordiformis*)<br>American beech (*Fagus grandifolia*)<br>White ash (*Fraxinus americana*)<br>American hophornbeam (*Ostrya virginiana*)<br>White spruce (*Picea glauca*)<br>Red pine (*Pinus resinosa*), White pine (*Pinus strobus*)<br>White poplar (*Populus grandidentata*), Quaking aspen (*Populus tremuloides*)<br>Black cherry (*Prunus serotina*)<br>Red oak (*Quercus rubra*)<br>American linden (*Tilia americana*)<br>Eastern hemlock-spruce (*Tsuga canadensis*)	unknown	[44]*

Supplementary Table 2. - continued

Continent	Country of occurrence	Host plant	Year of isolation	Reference
**America**	South Dakota	Ponderosa pine (*Pinus ponderosa*)	1995-1996	[45]*
	Washington	Sitka spruce (*Picea sitchensis*)<br>Douglas-fir (*Pseudotsuga menziesii*)<br>Salmonberry (*Rubus spectabilis*)<br>Scouler's willow (*Salix scouleriana*)<br>Western hemlock-spruce (*Tsuga heterophylla*)	1992-1993	[46]*
	Wisconsin	Balsam fir (*Abies balsamea*)<br>Sugar maple (*Acer saccharum*), Red maple (*Acer rubrum*)<br>Golden birch (*Betula alleghaniensis*), Paper birch (*Betula papyrifera*)<br>Black ash (*Fraxinus nigra*)<br>White spruce (*Picea glauca*), Sitka spruce (*Picea sitchensis*)<br>Jack pine (*Pinus banksiana*), Red pine (*Pinus resinosa*), White pine (*Pinus strobus*)<br>Quaking aspen (*Populus tremuloides*)<br>Red oak (*Quercus rubra*), Black oak (*Quercus velutina*)<br>Eastern hemlock-spruce (*Tsuga canadensis*)<br>Northern white-cedar (*Thuja occidentalis*)<br>Elms (*Ulmus* sp.)	1984-1994	[47]*
		Balsam fir (*Abies balsamea*)<br>Red-twigged shadbush (*Amelanchier sanguinea*)<br>Beaked hazelnut (*Corylus cornuta*)<br>Poplar (*Populus* sp.), Necklace poplar (*Populus deltoides*)<br>Red oak (*Quercus rubra*)<br>Wild lowbush (*Vaccinium angustifolium*)	1992	[48]*

Supplementary Table 2. - continued

Continent	Country of occurrence	Host plant	Year of isolation	Reference
**America**	Wisconsin	Balsam fir (*Abies balsamea*)<br>Sugar maple (*Acer saccharum*)<br>Paper birch (*Betula papyrifera*)<br>White spruce (*Picea glauca*)<br>Jack pine (*Pinus banksiana*), Red pine (*Pinus resinosa*), White pine (*Pinus strobus*)<br>Quaking aspen (*Populus tremuloides*)	unknown	[49]*
**Europe**	Norway	Norway spruce (*Picea abies*)	unknown	[16]
	Poland	Scots pine (*Pinus sylvestris*)	2005-2008	[19]

*originally reported as *A. ostoyae*

Supplementary Table 3. Geographical distribution of *Armillaria sinapina* based on epidemiology data

Continent	Country of occurrence	Host plant	Year of isolation	Reference
**America**	Alaska	Birch (*Betula* sp.)<br>White spruce (*Picea glauca*),<br>Willow (*Salix* sp.)<br>Quaking aspen (*Populus tremuloides*)<br>Mountain hemlock *(Tsuga mertensiana)*	2007	[50]
	British Columbia	*Picea glauca* × *Picea engelmannii*<br>Rocky Mountain Lodgepole Pine *(Pinus contorta var. latifolia)*<br>Douglas-fir (*Pseudotsuga menziesii*)	1997	[51]
	Canada	Silver maple (*Acer saccharinum*), Sugar maple (*Acer saccharum*)<br>Golden birch (*Betula alleghaniensis*), Paper birch (*Betula papyrifera*), Grey birch (*Betula populifolia*)<br>White pine (*Pinus strobus*)<br>White oak (*Quercus alba*)	1986	[32]
		Balsam fir (*Abies balsamea*)<br>Paper birch (*Betula papyrifera*)<br>White spruce (*Picea glauca*), Black spruce (*Picea mariana*)<br>Jack pine (*Pinus banksiana*), Lodgepole pine (*Pinus controta*)<br>Poplar (*Populus* sp.), Balsam poplar (*Populus balsamifera*), Quaking aspen (*Populus tremuloides*)<br>Douglas-fir (*Pseudotsuga menziesii*)	unknown	[33]
	Colorado	Subalpine fir (*Abies lasiocarpa*)<br>Western balsam bark beetle *(Dryocoetes confuses)*<br>Engelmann spruce *(Picea engelmannii)*<br>Quaking aspen (*Populus tremuloides*)	2014	[52]

Supplementary Table 3. - continued

Continent	Country of occurrence	Host plant	Year of isolation	Reference
**America**	Massachusetts	Red maple (*Acer rubrum*), Sugar maple (*Acer saccharum*)<br>American beech (*Fagus grandifolia*)<br>White ash (*Fraxinus americana*)	unknown	[36]
		Sugar maple (*Acer saccharum*)<br>Eastern hemlock-spruce (*Tsuga canadensis*)	unknown	[37]
	Michigan	Poplar (*Populus* sp.)	unknown	[53]
	New York	Balsam fir (*Abies balsamea*)<br>Red maple (*Acer rubrum*)<br>American beech (*Fagus grandifolia*)<br>White ash (*Fraxinus americana*)<br>Red spruce *(Picea rubens)*<br>Red pine (*Pinus resinosa*), White pine (*Pinus strobus)*<br>Black cherry (*Prunus serotina*)<br>Eastern hemlock-spruce (*Tsuga canadensis*)	1986-1989	[42]
	Ontario	Red maple (*Acer rubrum*), Sugar maple (*Acer saccharum*)<br>Yellow birch (*Betula alleghaniensis*), Paper birch (*Betula papyrifera*)<br>American beech (*Fagus grandifolia*)<br>White spruce (*Picea glauca*)<br>White pine (*Pinus strobus*)<br>White poplar (*Populus grandidentata*), Quaking aspen (*Populus tremuloides*)<br>Black cherry (*Prunus serotina*)<br>Red oak (*Quercus rubra*)<br>Eastern hemlock-spruce (*Tsuga canadensis*)	unknown	[44]

Supplementary Table 3. - continued

Continent	Country of occurrence	Host plant	Year of isolation	Reference
**America**	Wisconsin	Balsam fir (*Abies balsamea*)<br>Red maple (*Acer rubrum*)<br>Golden birch (*Betula alleghaniensis)*<br>Black ash (*Fraxinus nigra*)<br>Quaking aspen (*Populus tremuloides*)<br>White oak (*Quercus alba*)<br>Eastern hemlock-spruce (*Tsuga canadensis*)<br>Northern white-cedar (*Thuja occidentalis*)	1984-1994	[47]
**Asia**	China	Khingan fir *(Abies nephrolepis)*<br>Dahurian birch *(Betula dahurica)*, Erman's birch *(Betula ermanii)*. Japanese white birch *(Betula platyphylla)*<br>Olga Bay larch *(Larix olgensis)*<br>Pine *(Pinus* sp.*)*, Korean pine *(Pinus koraiensis)*<br>Mongolian oak (*Quercus mongolica*)<br>Willow (*Salix* sp.)	1996	[54]

Supplementary Table 4. Geographical distribution of *Armillaria gemina* based on epidemiology data

Continent	Country of occurrence	Host plant	Year of isolation	Reference
**America**	Canada	Sugar maple (*Acer saccharum*)	1987	[55]
	Massachusetts	Red maple (*Acer rubrum*), Sugar maple (*Acer saccharum*)<br>Golden birch (*Betula alleghaniensis)*<br>American beech (*Fagus grandifolia*)<br>Black cherry (*Prunus serotina*)	unknown	[36]
		Sugar maple (*Acer saccharum*)<br>Eastern hemlock-spruce (*Tsuga canadensis*)	unknown	[37]
	New York	Red maple (*Acer rubrum*), Sugar maple (*Acer saccharum*)<br>Golden birch (*Betula alleghaniensis*), Paper birch (*Betula papyrifera*)<br>American beech (*Fagus grandifolia*)<br>Scots pine (*Pinus sylvestris*)<br>Black cherry (*Prunus serotina*)<br>Red oak (*Quercus rubra*)	1986-1989	[42]
		Sugar maple (*Acer saccharum*)<br>American beech (*Fagus grandifolia*<br>Black cherry (*Prunus serotina*)	unknown	[43]
	Ontario	Red maple (*Acer rubrum*), Sugar maple (*Acer saccharum*)<br>Yellow birch (*Betula alleghaniensis*)<br>American beech (*Fagus grandifolia*)<br>American hophornbeam (*Ostrya virginiana*)<br>Black cherry (*Prunus serotina*)<br>Red oak (*Quercus rubra*)	unknown	[44]
	Pennsylvania	Red maple (*Acer rubrum*)	1995–1996	[56]

Supplementary Table 5. Geographical distribution of *Armillaria borealis* based on epidemiology data

Continent	Country of occurrence	Host plant	Year of isolation	Reference
**Asia**	Iran	Chestnut-leaved oak (*Quercus castaneifolia*)	1999-2001	[57]
**Europe**	Czech Republic	Norway spruce (*Picea abies*)	unknown	[5]
	England	Birch (*Betula* sp.)<br>European larch (*Larix decidua*)<br>Norway spruce (*Picea abies*), Sitka spruce (*Picea sitchensis*)<br>Scots pine (*Pinus sylvestris*)<br>Oak (*Quercus* sp.)	1978-1982	[58]
		Silver birch (*Betula pendula*)<br>Wild cherry (*Prunus avium*)	unknown	[8]
	Finland	Silver birch (*Betula pendula*), Downy birch (*Betula pubescens*)<br>Norway spruce (*Picea abies*)<br>Rowan (*Sorbus aucuparia*)	unknown	[59]
	Norway	Black alder (*Alnus glutinosa*), Gray alder (*Alnus incana*)<br>Birch (*Betula* sp.), Downy birch (*Betula pubescens*)<br>Norway spruce (*Picea abies*)<br>Lodgepole pine (*Pinus controta*), Scots pine (*Pinus sylvestris*)<br>Poplar (*Populus* sp.)<br>Oak (*Quercus* sp.)<br>Goat willow (*Salix caprea*)<br>Western hemlock-spruce (*Tsuga heterophylla*)	unknown	[16]
	Russia	Siberian fir (*Abies sibirica*)<br>Silver birch (*Betula pendula*)	2015	[60]

Supplementary Table 5. - continued

Continent	Country of occurrence	Host plant	Year of isolation	Reference
**Europe**	Russia	Norway spruce (*Picea abies*)<br>Siberian pine (*Pinus sibirica*)	2015	[60]
	Switzerland	Silver fir (*Abies alba*)<br>Norway maple (*Acer platanoides*)<br>Beech (*Fagus sylvatica*)<br>European larch (*Larix decidua*)<br>Norway spruce (*Picea abies*)<br>Mountain pine (*Pinus mugo*)	1992,<br>1999,<br>2010,<br>2011	[22]
		Mountain pine (*Pinus mugo*)	2003-2004	[24]
	Ukraine	Silver fir (*Abies alba*)	unknown	[27]

Supplementary Table 6. Geographical distribution of *Armillaria mellea* based on epidemiology data

Continent	Country of occurrence	Host plant	Year of isolation	Reference
**Africa**	Kenya	Tea tree (*Tea sinensis*)	unknown	[61]
		Tea plant (*Camellia sinensis*)<br>Chinese weeping cypress (*Cupressus funebris*)	unknown	[62]
	Sao Tome	Cacao tree (*Theobroma cacao*)	unknown	[61]
	South Africa	Buckeye/Chestnut (*Aesculus* sp.)<br>Silk tree (*Albizia* sp.)<br>Fig trees (*Ficus* sp.)<br>Hortensia (*Hydrengea* sp.)<br>Mulberries (*Morus* sp.)<br>Oak (*Quercus* sp.)<br>Crane flower (*Strelitzia* sp.)	1997	[63]
		Caperose (*Cliffortia ferruginea*)<br>Rock ash (*Ekebergia pterophylla*)<br>*Grevillea* sp.<br>Wild peach (*Kiggelaria africana*)<br>Silver tree (*Leucadendron argenteum*), Acacia-leaf conebush (*Leucadendron macowanii*), Peninsula conebush (*Leucadendron strobilium*)<br>Tree pincushion (*Leucospermum conocarpodendron*)<br>Satin bush (*Podalyria sericea*)<br>Narrow-leaf sugarbush (*Protea neriifolia*), Common sugarbush (*Protea repens*), Sawtooth sugarbush (*Protea sericea*)<br>Blossom tree (*Virgilia oroboides*)<br>Willowmore cedar (*Widdringtonia schwarzii*)	unknown	[64]

Supplementary Table 6. - continued

Continent	Country of occurrence	Host plant	Year of isolation	Reference
**America**		White oak (*Quercus alba*), Common red oak (*Quercus rubra*)	2004	[65]
	British Columbia	Big leaf maple (*Acer macrophyllum*)	1976-1977	[66]
	California	White fir (*Abies concolor*)<br>Incense cedar (*Libocedrus decurrens*)<br>Pine (*Pinus* sp.), Ponderosa pine (*Pinus ponderosa*)<br>Almond (*Prunus dulcis*)<br>Coast live oak (*Quercus agrifolia*), California black oak (*Quercus kelloggii*), Valley oak (*Quercus lobata*)<br>California laurel (*Umbellularia californica*)	1988-1989	[67]
		Almond (*Prunus dulcis*), Peach (*Prunus persica*)	unknown	[68]
		Maple (*Acer* sp.)<br>Silk tree (*Albizia* sp.)<br>White alder (*Alnus rhombifolia*)<br>Mandrone (*Arbutus menziesii*)<br>Birch (*Betula* sp.)<br>Incense cedar (*Calocedrus decurrens*)<br>*Camellia* sp.<br>Chestnut (*Castanea* sp.)<br>Buckbrush (*Ceanothus* sp.)<br>Deodar cedar (*Cedrus deodara*)<br>Camphorwood (*Cinnamomum camphora*)<br>*Citrus* sp.<br>*Cotoneaster* sp.<br>Monterey cypress (*Cupressus macrocarpa*)<br>*Cycas* sp.	unknown	[69]

Supplementary Table 6. - continued

Continent	Country of occurrence	Host plant	Year of isolation	Reference
**America**	California	Loquat (*Eriobotrya japonica*)<br>*Eucalyptus* sp.<br>Box leaf hebe (*Hebe buxifolia*)<br>Toyon (*Heteromeles arbutifolia*)<br>Holly (*Ilex* sp.)<br>Eastern black walnut (*Juglans nigra*)<br>Juniper (*Juniperus* sp.)<br>Tea tree (*Leptospermum* sp.)<br>Wild privet (*Ligustrum vulgare*)<br>Sweetgum (*Liquidambar* sp.)<br>*Magnolia* sp.<br>Apple (*Malus* sp.)<br>Banana (*Musa* sp.)<br>Oleander (*Nerium oleander*)<br>Boldo (*Peumus boldus*)<br>Pine (*Pinus* sp.)<br>*Prunus* sp.<br>Douglas-fir (*Pseudotsuga menziesii*)<br>Firethorn (*Pyracantha* sp.)<br>Common pear (*Pyrus communis*)<br>Oak (*Quercus* sp.), Coast live oak (*Quercus agrifolia*), California black oak (*Quercus kelloggii*), Valley oak (*Quercus lobata*)<br>Buckthorns (*Rhamnus* sp.)<br>*Rhododendron* sp.<br>*Rosa* sp.<br>Willow (*Salix* sp.)<br>Peruvian pepper (*Schinus molle*)<br>California redwood (*Sequoia sempervirens*)	unknown	[69]

Supplementary Table 6. - continued

Continent	Country of occurrence	Host plant	Year of isolation	Reference
**America**	California	Giant sequoia (*Sequoiadendron giganteum*)<br>Common lilac (*Syringa vulgaris*)<br>Elms (*Ulmus* sp.)<br>California laurel (*Umbellularia californica*)<br>Sandankwa viburnum (*Viburnum suspensum*)<br>*Vitis* sp.<br>California palm (*Washingtonia filifera*)	unknown	[69]
		Mandrone (*Arbutus menziesii*)<br>Boxleaf azara (*Azara microphylla*)<br>*Callistemon* sp., River bottlebrush (*Callistemon sieberi*)<br>*Camellia* sp., Japanese camellia (*Camellia japonica*)<br>Monterey cypress (*Cupressus macrocarpa*)<br>*Eucalyptus* sp.<br>Toyon (*Heteromeles arbutifolia*)<br>Holly (*Ilex* sp.)<br>Juniper (*Juniperus* sp.)<br>Boldo (*Peumus boldus*)<br>Pine (*Pinus* sp.), Ponderosa pine (*Pinus ponderosa*), Monterey pine (*Pinus radiata*)<br>*Pseudotsuga* sp.<br>Oak (*Quercus* sp.), Netleaf oak (*Quercus rugosa*), Interior live oak (*Quercus wislizenii*)<br>Black locust (*Robinia pseudoacacia*)<br>*Rhododendron* sp.<br>Willow (*Salix* sp.)<br>Montezuma cypress (*Taxodium mucronatum*)	2001-2002	[70]
	Connecticut	Sugar maple (*Acer saccharum*)<br>Black oak (*Quercus velutina*)	1976-1977	[66]

Supplementary Table 6. - continued

Continent	Country of occurrence	Host plant	Year of isolation	Reference
**America**	Idaho	Grand fir (*Abies grandis*)	1976-1977	[66]
	Massachusetts	Red maple (*Acer rubrum*)	1976-1977	[66]
		Lilac (*Syringa* sp.)	unknown	[71]
		Sweet birch (*Betula lenta*), Paper birch (*Betula papyrifera*)<br>American chestnut (*Castanea dentata*)<br>Pitch pine (*Pinus rigida*), White pine (*Pinus strobus*)<br>Black cherry (*Prunus serotina*)<br>White oak (*Quercus alba*), Scarlet oak (*Quercus coccinea*), Black oak (*Quercus velutina*)	unknown	[36]
	Mexico	Peach (*Prunus persica*)	2009-2011	[72]
	Michigan	Sugar maple (*Acer saccharum*)<br>Yellow birch (*Betula alleghaniensis*)	unknown	[73]
		American beech (*Fagus grandifolia*)<br>American tulip tree (*Liriodendron tulipifera*)<br>Black cherry (*Prunus serotina*)<br>White oak (*Quercus alba*)	1976-1977	[66]
	Missouri	Hickory (*Carya* sp.)<br>Pine *(Pinus* sp.*)*<br>White oak (*Quercus alba*), Red oak (*Quercus rubra*)	1993-1995	[74]
		White alder (*Nyssa sylvatica*)<br>Scarlet oak (*Quercus coccinea*), Blackjack oak (*Quercus marilandica*), Black oak (*Quercus velutina*)	1993-1997	[75]

Supplementary Table 6. - continued

Continent	Country of occurrence	Host plant	Year of isolation	Reference
**America**	New York	Maple (*Acer* sp.), Sugar maple (*Acer saccharum*)<br>Yellow birch (*Betula alleghaniensis*)<br>Flowering dogwood (*Cornus florida*)<br>Ash (*Fraxinus* sp.)<br>American tulip tree (*Liriodendron tulipifera*)<br>Black cherry (*Prunus serotina*)	1976-1977	[66]
		White oak (*Quercus alba*)	1986-1989	[42]
	Ontario	Paper birch (*Betula papyrifera*)	1976-1977	[66]
		Paper birch (*Betula papyrifera*)<br>American beech (*Fagus grandifolia*)<br>Red oak (*Quercus rubra*)	unknown	[44]
	Pennsylvania	Red maple (*Acer rubrum*)	1995-1996	[56]
	Vermont	Balsam fir (*Abies balsamea*)<br>Sugar maple (*Acer saccharum*)<br>Birch (*Betula* sp.), Yellow birch (*Betula alleghaniensis*)<br>American beech (*Fagus grandifolia*)<br>White spruce (*Picea glauca*)<br>Lodgepole pine (*Pinus contorta*), White pine (*Pinus strobus*)<br>White oak (*Quercus alba*), Red oak (*Quercus rubra*)	unknown	[73]
	Washington	Grand fir (*Abies grandis*)<br>Lodgepole pine (*Pinus contorta*), Ponderosa pine (*Pinus ponderosa*)	1976-1977	[66]

Supplementary Table 6. - continued

Continent	Country of occurrence	Host plant	Year of isolation	Reference
**America**	Wisconsin	Sugar maple (*Acer saccharum*)	unknown	[73]
		Sugar maple (*Acer saccharum*)<br>White oak (*Quercus alba*), Red oak (*Quercus rubra*), Black oak (*Quercus velutina*)	1984-1994	[47]
**Asia**	China	*Keteleeria* sp.<br>Sikang pine (*Pinus densata*)	1996	[54]
	Iran	Maple (*Acer* sp.)<br>Oriental beech (*Fagus orientalis*)<br>Persian ironwood (*Parrotia persica)*<br>Oriental plane (*Platanus orientalis*)<br>*Prunus* sp., Almond (*Prunus dulcis*)	1999-2001	[57]
		Bitter orange (*Citrus aurantium*)<br>Oriental beech (*Fagus orientalis*)<br>Persian walnut (*Juglans regia*)<br>Persian ironwood (*Parrotia persica*)<br>Oriental plane (*Platanus orientalis*)<br>Ansu apricot (*Prunus armeniaca*), Almond (*Prunus dulcis*)<br>Common pear (*Pyrus communis*)	2006-2008	[76]
	Japan	Painted maple (*Acer pictum* ssp. *mono*)<br>Japanese ash (*Fraxinus lanuginosa*)<br>Mongolian oak (*Quercus mongolica* var. *grosseserrata*)	1993-1994	[77]

Supplementary Table 6. - continued

Continent	Country of occurrence	Host plant	Year of isolation	Reference
**Asia**	Japan	Bitter orange (*Citrus aurantium*)<br>Japanese cypress (*Chamaecyparis obtusa*)<br>Japanese red pine (*Pinus densiflora*), Japanese black pine (*Pinus thunbergii*)	unknown	[78]
		Japanese horse-chestnut (*Aesculus turbinata*)<br>Katsura (*Cercidiphyllum japonicum*)<br>Japanese cypress (*Chamaecyparis obtusa*)<br>Japanese red pine (*Pinus densiflora*)<br>Japanese pear (*Pyrus pyrifolia*)<br>Sawtooth oak (*Quercus acutissima*), Jolcham oak (*Quercus serrata*)	unknown	[79]
		Japanese cypress (*Chamaecyparis obtusa*)<br>Japanese black pine (*Pinus thunbergii*)	1976-2007	[2]
**Europe**	Albania	Silver fir (*Abies alba*), Greek fir (*Abies borisi-regis*),<br>Sycamore (*Acer pseudoplatanus*)<br>Black alder (*Alnus glutinosa*), Gray alder (*Alnus incana*), Green alder (*Alnus viridis*)<br>Silver birch (*Betula pendula*)<br>Hornbeam (*Carpinus betulus*)<br>Sweet chestnut (*Castanea sativa*)<br>Rangpur (*Citrus limonia*), Sweet orange (*Citrus sinensis*)<br>Hazelnut (*Corylus avellana*)<br>Common hawthorn (*Crategus monogyna*)<br>Cypress (*Cupressus sempervirens*)<br>Beech (*Fagus sylvatica*)<br>Common fig (*Ficus carica*)<br>Common ash (*Fraxinus excelsior*)<br>Persian walnut (*Juglans regia*)	1990-2006	[3]

Supplementary Table 6. - continued

Continent	Country of occurrence	Host plant	Year of isolation	Reference
**Europe**		Common juniper (*Juniperus communis*)<br>Apple (*Malus* sp.), European crabapple (*Malus sylvestris*)<br>White mulberry (*Morus alba*), Black mulberry (*Morus nigra*)<br>Oleander (*Nerium oleander*)<br>Olive (*Olea* sp.), European olive (*Olea europaea*)<br>Hop hornbeam (*Ostrya carpinifolia*)<br>Aleppo pine (*Pinus halepensis*), Austrian pine (*Pinus nigra*)<br>Oriental plane (*Platanus orientalis*)<br>*Prunus* sp., Wild cherry (*Prunus avium*), Common plum (*Prunus domestica*), Almond (*Prunus dulcis*), Peach (*Prunus persica*), Black cherry (*Prunus serotina*)<br>Pear (*Pyrus* sp.), Common pear (*Pyrus communis*)<br>Austrian oak (*Quercus cerris*), Hungarian oak (*Quercus frainetto*), Holm oak (*Quercus ilex*), Sessile oak (*Quercus petraea*), Pubescent oak (*Quercus pubescens*), Common oak (*Quercus robur*), Red oak (*Quercus rubra*)<br>Black locust (*Robinia pseudoacacia*)<br>Common willow (*Salix alba*), Green weeping willow (*Salix babylonica*)<br>Northern white-cedar (*Thuja occidentalis*), Chinese thuja (*Thuja orientalis*)<br>Silver lime (*Tilia tomentosa*)<br>Wych elm (*Ulmus glabra*), English elm (*Ulmus procera*)<br>*Vitis* sp., Common grape vine (*Vitis vinifera*)	1990-2006	[3]
	Austria	Silver fir (*Abies alba*), Grand fir (*Abies grandis*)<br>Norway spruce (*Picea abies*)<br>Douglas fir (*Pseudotsuga menziesii*)	unknown	[80]
	Bulgaria	Austrian oak (*Quercus cerris*), Hungarian oak (*Quercus frainetto*), Sessile oak (*Quercus petraea*), Red oak (*Quercus rubra*)	1991-1999	[81]

Supplementary Table 6. - continued

Continent	Country of occurrence	Host plant	Year of isolation	Reference
**Europe**	Czech Republic	Black alder (*Alnus glutinosa*)	unknown	[5]
	England	Norway spruce (*Picea abies*)<br>Scots pine (*Pinus sylvestris*), Corsican pine *(Pinus nigra)*<br>Douglas-fir (*Pseudotsuga menziesii*)	unknown	[6,7]
	England	Field maple (*Acer campestre*), Big leaf maple (*Acer macrophyllum*), Italian maple *(Acer opalus),* Sycamore (*Acer pseudoplatanus*), Silver maple (*Acer saccharinum*),<br>Horse chestnut (*Aesculus hippocastanum)*<br>Black alder (*Alnus glutinosa*), Gray alder (*Alnus incana*)<br>Alternate-leaved butterfly-bush *(Buddleja alternifolia)*<br>Incense cedar (*Calocedrus decurrens*)<br>Atlas cedar (*Cedrus atlantica*), Lebanon cedar *(Cedrus libani)*<br>Lawson cypress (*Chamaecyparis lawsoniana*)<br>Pacific dogwood *(Cornus nuttallii)*<br>Rockspray cotoneaster *(Cotoneaster horizontalis),* Late cotoneaster (*Cotoneaster lacteus)*<br>Common hawthorn (*Crataegus monogyna*)<br>Leyland cypress *(Cupressocyparis leylandii)*<br>Smooth Arizona Cypress *(Cupressus glabra)*<br>Japanese cedar (*Cryptomeria japonica)*<br>*Deutzia* sp.<br>Weeping forsythia *(Forsythia suspensa)*<br>Common ash (*Fraxinus excelsior*)<br>Common holly (*Ilex aquifolium*)<br>Persian walnut (*Juglans regia*)<br>Japanese marigold bush *(Kerria japonica)*<br>Beauty bush *(Kolkwitzia amahilis)*<br>Common laburnum *(Laburnum anagyroides)*<br>European larch (*Larix decidua*)	unknown	[8]

Supplementary Table 6. - continued

Continent	Country of occurrence	Host plant	Year of isolation	Reference
**Europe**	England	Wild privet (*Ligustrum vulgare*)<br>American tulip tree (*Liriodendron tulipifera*)<br>Oregon grape-holly *(Mahonia japonica)*<br>Apple (*Malus pumila*), European crabapple (*Malus sylvestris*)<br>Black mulberry (*Morus nigra*)<br>European olive (*Olea europaea*)<br>English dogwood (*Philadelphus coronarius)*<br>*Pinus nigra* subsp. *laricio,* Maritime pine (*Pinus pinaster*), Stone pine (*Pinus pinea*), Scots pine (*Pinus sylvestris*), Bhutan pine (*Pinus wallichiana*)<br>London plane *(Platanus acerifolia)*<br>Poplar (*Populus* sp.)<br>*Prunus* sp., Wild cherry (*Prunus avium*), Cherry plum (*Prunus cerasifera, Prunus cerasifera* var. *pissardii),* Peach (*Prunus persica*)<br>Douglas-fir (*Pseudotsuga menziesii*)<br>Firethorn (*Pyracantha* sp.)<br>Common pear (*Pyrus communis*)<br>Common oak (*Quercus robur*)<br>Flowering currant *(Ribes sanguineum)*<br>*Rosa* sp<br>Willow (*Salix* sp.), Common willow (*Salix alba*), Coral bark willow *(Salix alba* ssp. *vitellina),* Green weeping willow (*Salix babylonica*), Goat willow (*Salix caprea*)<br>Giant sequoia (*Sequoiadendron giganteum*)<br>*Sorbus* sp., Rowan (*Sorbus aucuparia*)<br>Common lilac (*Syringa vulgaris*)<br>Western red cedar (*Thuja plicata*)<br>Linden (*Tilia* sp.)<br>Western hemlock-spruce (*Tsuga heterophylla*)<br>Elm *(Ulmus* sp.)<br>*Viburnum* sp., Viburnum *(Viburnum farreri),* Laurustinus (*Viburnum tinus*)	unknown	[8]

Supplementary Table 6. - continued

Continent	Country of occurrence	Host plant	Year of isolation	Reference
**Europe**	England	Birch (*Betula* sp.)<br>*Rosa* sp.<br>Lilac (*Syringa* sp.)	1997	[82]
		Privet (*Ligustrum* sp.)	unknown	[83]
		Buckeye/Chestnut (*Aesculus* sp.)<br>Akebia (*Akebia* sp.)<br>Birch (*Betula* sp.)<br>Silk cotton tree (*Bombax* sp.)<br>*Brachyglottis* sp.<br>Palm (*Chamaerops* sp.)<br>*Cordyline* sp.<br>Smoketree (*Cotinus* sp.)<br>Pearl bush (*Exochorda* sp.)<br>Witch hazels (*Hamamelis* sp.)<br>Privet (*Ligustrum* sp.)<br>*Linnaea* sp.<br>Mercuries (*Mercurialis* sp.)<br>*Miosa* sp.<br>Beardtongues (*Penstemon* sp.)<br>*Prunus* sp.<br>*Rhododendron* sp.<br>*Skimmia* sp.<br>Rowan (*Sorbus* sp.)<br>*Stachyurus* sp.	2004-2007	[9]

Supplementary Table 6. - continued

Continent	Country of occurrence	Host plant	Year of isolation	Reference
**Europe**		Boxwood *(Buxus* sp.)<br>Leyland cypress *(Cupressocyparis leylandii)*<br>Spindle *(Euonymous* sp.)<br>Privet (*Ligustrum* sp.), Wild privet (*Ligustrum vulgare*)<br>*Prunus* sp., Ansu apricot (*Prunus armeniaca*), Common plum (*Prunus domestica*), Damson plum *(Prunus institia),* Cherry laurel *(Prunus laurcerasus),* Portugal laurel *(Prunus lusitanica)*<br>*Rhododendron* sp.<br>Sweet box *(Sarcococca* sp.)<br>*Viburnum* sp.	2004-2007<br>2017	[10]
	France	Peach (*Prunus persica*)	1969	[84]
		Common oak (*Quercus robur*)	1975	[85]
		Beech (*Fagus* sp.)	1990	[11]
		Wattle *(Acacia* sp.), Silver wattle *(Acacia dealbata)*<br>Norway maple (*Acer platanoides*), Sycamore (*Acer pseudoplatanus*)<br>Golden kiwifruit (*Actinidia chinensis*)<br>Horse chestnut *(Aesculus hippocastanum)*<br>Giant cane *(Arundo donax)*<br>Silver birch (*Betula pendula*)<br>Atlas cedar (*Cedrus atlantica*), Deodar cedar (*Cedrus deodara*)<br>European nettle tree *(Celtis australis)*<br>Carob *(Ceratonia siliqua)*<br>Lawson cypress (*Chamaecyparis lawsoniana*)<br>Mums *(Chrysanthemum* sp.*)*<br>Grey-leaved cistus *(Cistus albidus)*	unknown	[8]

Supplementary Table 6. - continued

Continent	Country of occurrence	Host plant	Year of isolation	Reference
**Europe**	France	Bitter orange (*Citrus aurantium*)<br>Hazelnut (*Corylus avellana*)<br>Rockspray cotoneaster *(Cotoneaster horizontalis)*<br>Leyland cypress *(Cupressocyparis leylandii)*<br>Arizona cypress *(Cupressus arizonica),* Smooth Arizona Cypress *(Cupressus glabra),* Cypress (*Cupressus sempervirens*)<br>Quince *(Cydonia vulgaris)*<br>Artichoke *(Cynara scolymus)*<br>Loquat (*Eriobotrya japonica*)<br>*Eucalyptus* sp., Mountain gum *(Eucalyptus dalrympleana)*<br>Beech (*Fagus sylvatica*)<br>Common fig (*Ficus carica*)<br>Common ash (*Fraxinus excelsior*)<br>Common ivy *(Hedera helix)*<br>Persian walnut (*Juglans regia*)<br>European larch (*Larix decidua*)<br>Wild privet (*Ligustrum vulgare*)<br>American tulip tree (*Liriodendron tulipifera*)<br>Apple (*Malus pumila*)<br>European olive (*Olea europaea*)<br>Virginia creeper *(Parthenocissus quinquefolia)*<br>London plane *(Platanus acerifolia)*<br>Ansu apricot (*Prunus armeniaca*), Wild cherry (*Prunus avium*), Cherry plum *(Prunus cerasifera),* Common plum (*Prunus domestica*), Almond (*Prunus dulcis*), Mahaleb cherry *(Prunus mahaleb),* Peach (*Prunus persica*)<br>Douglas-fir (*Pseudotsuga menziesii*)<br>Firethorn (*Pyracantha* sp.)<br>Common pear (*Pyrus communis*)	unknown	[8]

Supplementary Table 6. - continued

Continent	Country of occurrence	Host plant	Year of isolation	Reference
**Europe**	France	Sessile oak (*Quercus petraea*), Common oak (*Quercus robur*), Red oak (*Quercus rubra*), Cork oak (*Quercus suber*)<br>Blackcurrant *(Ribes nigrum)*<br>*Rosa* sp.<br>Willow (*Salix* sp.)<br>Chequers *(Sorbus torminalis)*<br>Crane flower *(Strelitzia reginae*<br>Cedar *(Thuja* sp.), Northern white-cedar (*Thuja occidentalis*),<br>Linden (*Tilia* sp.)<br>Laurustinus (*Viburnum tinus*)<br>*Vitis* sp., Common grape vine (*Vitis vinifera*)	unknown	[8]
	Germany	Sycamore (*Acer pseudoplatanus*)	1997	[86]
	Greece	Greek fir (*Abies borisi-regis*), Bulgarian fir (*Abies cephalonica*)<br>Golden kiwifruit (*Actinidia chinensis*)<br>Hornbeam (*Carpinus betulus*)<br>Sweet chestnut (*Castanea sativa*)<br>Atlas cedar (*Cedrus atlantica*)<br>Pink rock-rose (*Cistus creticus*)<br>Lemon (*Citrus limon*)<br>Hazelnut (*Corylus avellana*) Balkan beech (*Fagus moesiaca*), Beech (*Fagus sylvatica*)<br>Common ash (*Fraxinus excelsior*)<br>French broom (*Genista monspessulana*)<br>Persian walnut (*Juglans regia*)<br>Common juniper (*Juniperus communis*)<br>European crabapple (*Malus sylvestris*)<br>White mulberry (*Morus alba*)	1991-1996	[14]

Supplementary Table 6. - continued

Continent	Country of occurrence	Host plant	Year of isolation	Reference
**Europe**	Greece	Oleander (*Nerium oleander*)<br>European olive (*Olea europaea*)<br>Aleppo pine (*Pinus halepensis*), Austrian pine (*Pinus nigra*)<br>Oriental plane (*Platanus orientalis*)<br>Wild cherry (*Prunus avium*), Common plum (*Prunus domestica*), Almond (*Prunus dulcis*), Peach (*Prunus persica*)<br>Common pear (*Pyrus communis*)<br>Hungarian oak (*Quercus frainetto*)<br>Chinese thuja (*Thuja orientalis*)<br>Laurustinus (*Viburnum tinus*)<br>Common grape vine (*Vitis vinifera*)	1991-1996	[14]
	Hungary	Oak (*Quercus* sp.)	2016	[4]
	Italy	Silver fir (*Abies alba*)<br>Field maple (*Acer campestre*)<br>Golden kiwifruit (*Actinidia chinensis*)<br>Horse chestnut (*Aesculus hippocastanum)*<br>Gray alder (*Alnus incana*)<br>Hornbeam (*Carpinus betulus*)<br>Sweet chestnut (*Castanea sativa*)<br>Atlas cedar (*Cedrus atlantica*)<br>European nettle tree (*Celtis australis)*<br>Lawson cypress (*Chamaecyparis lawsoniana)*<br>Bitter orange (*Citrus aurantium*), Lime (*Citrus limonum)*<br>Hazelnut (*Corylus avellana*)<br>Arizona cypress *(Cupressus arizonica)*, Monterey cypress (*Cupressus macrocarpa*), Cypress (*Cupressus sempervirens*)	unknown	[8]

Supplementary Table 6. - continued

Continent	Country of occurrence	Host plant	Year of isolation	Reference
**Europe**	Italy	*Eucalyptus* sp.<br>Beech (*Fagus sylvatica*)<br>Common fig (*Ficus carica*)<br>Common ash (*Fraxinus excelsior*), South European flowering ash *(Fraxinus ornus)*<br>Bridal broom *(Genista monosperma)*<br>Persian walnut (*Juglans regia*)<br>European larch (*Larix decidua*)<br>Bay tree *(Laurus nobilis)*<br>Apple (*Malus pumila*)<br>White mulberry (*Morus alba*), Black mulberry (*Morus nigra*)<br>European olive (*Olea europaea*)<br>Prickly pear *(Opuntia ficus-indica)*<br>Hop hornbeam (*Ostrya carpinifolia*)<br>Norway spruce (*Picea abies*)<br>Aleppo pine (*Pinus halepensis*), *Pinus nigra* subsp. *laricio,* Maritime pine (*Pinus pinaster*), Stone pine (*Pinus pinea*)<br>London plane *(Platanus acerifolia)*<br>Poplar (*Populus* sp.)<br>Ansu apricot (*Prunus armeniaca*), Wild cherry (*Prunus avium*), Cherry plum *(Prunus cerasifera),* Common plum (*Prunus domestica*), Almond (*Prunus dulcis*), Peach (*Prunus persica*)<br>Douglas fir (*Pseudotsuga menziesii*)<br>Common pear (*Pyrus communis*)<br>Turkey oak (*Quercus cerris*), Hungarian oak (*Quercus frainetto),* Holm oak (*Quercus ilex*), Sessile oak (*Quercus petraea*), Common oak (*Quercus robur*), Cork oak (*Quercus suber*)<br>Black locust (*Robinia pseudoacacia*)<br>*Rosa* sp.<br>Willow (*Salix* sp.)<br>European elder (*Sambucus nigra)*	unknown	[8]

Supplementary Table 6. - continued

Continent	Country of occurrence	Host plant	Year of isolation	Reference
**Europe**	Italy	Peruvian pepper (*Schinus molle*)<br>Spanish broom *(Spartium junceum)*<br>Linden (*Tilia* sp.)<br>Elms (*Ulmus* sp.)<br>*Vitis* sp., Common grape vine (*Vitis vinifera*)	unknown	[8]
		House holly-fern (*Cyrtomium falcatum*)	1999	[87]
		Common grape vine (*Vitis vinifera*)	2000-2004	[88]
		Pineapple palm (*Phoenix canariensis*)	2006	[89]
	Lithuania	Small-leaved lime (*Tilia cordata*)	2005-2011	[90]
	Netherlands	Field maple (*Acer campestre*)<br>Gray alder (*Alnus incana*)<br>Birch (*Betula* sp.)<br>Hazelnut (*Corylus avellana*)<br>Beech (*Fagus sylvatica*)<br>Common ash (*Fraxinus excelsior*)<br>Apple (*Malus* sp.)<br>Silver poplar (*Populus alba*), Aspen (*Populus tremula*)<br>Wild cherry (*Prunus avium*)<br>Common pear (*Pyrus communis*)<br>Common oak (*Quercus robur*)<br>Black locust (*Robinia pseudoacacia*)<br>Common willow (*Salix alba*), Common sallow (*Salix cinerea*)<br>Linden (*Tilia* sp.)	1983-1992	[15]

Supplementary Table 6. - continued

Continent	Country of occurrence	Host plant	Year of isolation	Reference
**Europe**	Portugal	Cork oak (*Quercus suber*)	unknown	[91]
	Serbia	Hornbeam (*Carpinus betulus*)<br>Sessile oak (*Quercus petraea*)	unknown	[92]
		Hornbeam (*Carpinus betulus*), Oriental hornbeam (*Carpinus orientalis*)<br>Balkan Beech (*Fagus moesiaca*)<br>Norway spruce (*Picea abies*), Purkyne Serbian Spruce (*Picea omorika*)<br>Pine (*Pinus* sp.)<br>Hungarian oak (*Quercus frainetto*), Sessile oak (*Quercus petraea*), Turkey oak (*Quercus cerris*)	2002-2007	[20]
	Spain	Common grape vine (*Vitis vinifera*)	1995-1997	[93]
		Common grape vine (*Vitis vinifera*)	unknown	[94]
		Scots pine (*Pinus sylvestris*)	unknown	[95]
		Lawson cypress (*Chamaecyparis lawsoniana*)<br>Common ash (*Fraxinus excelsior*)<br>Oak (*Quercus* sp.), Pyrenean oak (*Quercus pyrenaica*)<br>Monterey pine (*Pinus radiata*)	unknown	[96]

Supplementary Table 7. Geographical distribution of *Armillaria gallica* based on epidemiology data

Continent	Country of occurrence	Host plant	Year of isolation	Reference
**America**	Arizona	Ponderosa pine (*Pinus ponderosa*)<br>Douglas-fir (*Pseudotsuga menziesii*)<br>Gambel oak (*Quercus gambelii*)	2010	[97]
	Arkansas	White oak (*Quercus alba*), Common red oak (*Quercus rubra*)	2004	[65]
	California	White fir (*Abies concolor*), Red fir (*Abies magnifica*)<br>Big leaf maple (*Acer macrophyllum*)<br>California buckeye (*Aesculus californica*)<br>Red alder (*Alnus rubra*)<br>Mandrone (*Arbutus menziesii*)<br>Manzanita (*Arctostaphylos* sp.)<br>Incense cedar (*Calocedrus decurrens*)<br>Dogwoods (*Cornus* sp.)<br>Tanoak (*Lithocarpus densiflorus*)<br>Sugar pine (*Pinus lambertiana*), Ponderosa pine (*Pinus ponderosa*)<br>Bitter-berry (*Prunus virginiana*)<br>Douglas-fir (*Pseudotsuga menziesii*)<br>Coast live oak (*Quercus agrifolia*), California black oak (*Quercus kelogii*), Valley oak (*Quercus lobata*)<br>California redwood (*Sequoia sempervirens*)<br>California laurel (*Umbellularia californica*)	unknown	[69]
	Georgia	*Rhododendron* sp.<br>Red oak (*Quercus rubra*)	2017	[98]
	Hawaii	Monterey pine (*Pinus radiata*), Loblolly pine (*Pinus taeda*)<br>Methley plum (*Prunus cerasifera* × *Prunus salicina*)<br>Māmane (*Sophora chrysophylla*)	2005	[99]

Supplementary Table 7. –continued

Continent	Country of occurrence	Host plant	Year of isolation	Reference
**America**	Hawaii	Koa (*Acacia koa*)<br>'Öhi'a lehua (*Metrosideros polymorpha*)	2015	[100]
	Massachusetts	Red maple (*Acer rubrum*)<br>White ash (*Fraxinus americana*)<br>Spruce (*Picea* sp.)<br>Common lilac (*Syringa vulgaris*)	1988	[35]
	Massachusetts	Red maple (*Acer rubrum*), Sugar maple (*Acer saccharum*)<br>Yellow birch (*Betula alleghaniensis*), Sweet birch (*Betula lenta*), Paper birch (*Betula papyrifera*)<br>Shagbark hickory (*Carya ovata*)<br>American beech (*Fagus grandifolia*)<br>White ash (*Fraxinus americana*)<br>White pine (*Pinus strobus*)<br>Quaking aspen (*Populus tremuloides*)<br>White oak (*Quercus alba*), Chestnut oak (*Quercus prinus*), Common red oak (*Quercus rubra*), Black oak (*Quercus velutina*)<br>American linden (*Tilia americana*)	unknown	[36]
		Red maple (*Acer rubrum*)<br>Yellow birch (*Betula alleghaniensis*), Paper birch (*Betula papyrifera*)<br>American beech (*Fagus grandifolia*)<br>White ash (*Fraxinus americana*)<br>Pitch pine (*Pinus rigida*), White pine (*Pinus strobus*)<br>White poplar (*Populus grandidentata*)<br>Black cherry (*Prunus serotina*)<br>White oak (*Quercus alba*), Swamp white oak (*Quercus bicolor*), Common red oak (*Quercus rubra*), Black oak (*Quercus velutina*)	2010	[37]

Supplementary Table 7. –continued

Continent	Country of occurrence	Host plant	Year of isolation	Reference
**America**	Mexico	Sacred fir (*Abies religiosa*)<br>Alder (*Alnus* sp., *Alnus acuminata*)<br>Texas madrone (*Arbutus xalapensis*)<br>American hornbeam (*Carpinus caroliniana*)<br>Loquat (*Eriobotrya japonica*)<br>American sweetgum (*Liquidambar styraciflua*)<br>Johnnyberry (*Miconia mexicana*)<br>Mexican plane (*Platanus mexicana*)<br>Groundsel (*Roldana* sp.)<br>*Senecio* sp.<br>Xalapa oak (*Quercus xalapensis*)	2007	[101]
		Orchard apple *(Malus domestica)*<br>Pino blanco *(Pinus devoniana),* Smooth-bark Mexican pine *(Pinus pseudostrobus),* Teocote *(Pinus teocote)*<br>*Quercus jonesii,* American turkey oak *(Quercus laevis)*	2019	[102]
	Missouri	Hickory (*Carya* sp.)<br>Pine *(Pinus* sp.*)*<br>White oak (*Quercus alba*), Red oak (*Quercus rubra*)	1993-1995	[74]
		White alder (*Nyssa sylvatica*)<br>Scarlet oak (*Quercus coccinea*), Blackjack oak (*Quercus marilandica*), Black oak (*Quercus velutina*)	1993-1997	[75]
	Nebraska	Paper birch (*Betula papyrifera*)<br>White ash (*Fraxinus americana*)<br>Ponderosa pine (*Pinus ponderosa*)<br>Necklace poplar (*Populus deltoides*)<br>Bur oak (*Quercus macrocarpa*)<br>American linden (*Tilia americana*)<br>American elm (*Ulmus americana*)	unknown	[103]

Supplementary Table 7. –continued

Continent	Country of occurrence	Host plant	Year of isolation	Reference
**America**	New York	Balsam fir (*Abies balsamea*)<br>Red maple (*Acer rubrum*), Sugar maple (*Acer saccharum*)<br>Golden birch (*Betula alleghaniensis*), Paper birch (*Betula papyrifera*)<br>American beech (*Fagus grandifolia*)<br>White ash (*Fraxinus americana*)<br>Red pine (*Pinus resinosa*), White pine (*Pinus strobus*), Scots pine (*Pinus sylvestris*)<br>Black cherry (*Prunus serotina*)<br>White oak (*Quercus alba*), Red oak (*Quercus rubra*)<br>American linden (*Tilia americana*)<br>Eastern hemlock-spruce (*Tsuga canadensis*)	1986-1989	[42]
		Sugar maple (*Acer saccharum*)<br>American beech (*Fagus grandifolia)*<br>Black cherry (*Prunus serotina*)	unknown	[43]
	Ontario	Red maple (*Acer rubrum*), Silver maple (*Acer saccharinum*), Sugar maple (*Acer saccharum*)<br>Yellow birch (*Betula alleghaniensis*), Paper birch (*Betula papyrifera*)<br>American hornbeam (*Carpinus caroliniana*)<br>Bitternut hickory (*Carya cordiformis*), Shagbark hickory (*Carya ovata*)<br>American beech (*Fagus grandifolia*)<br>White ash (*Fraxinus americana*), Green ash (*Fraxinus pennsylvanica*)<br>Butternut (*Juglans cinerea*), Eastern black walnut (*Juglans nigra*)<br>Tulip tree (*Liriodendron tulipifera*)<br>American hophornbeam (*Ostrya virginiana*)<br>Jack pine (*Pinus banksiana*), Red pine (*Pinus resinosa*), White pine (*Pinus strobus*)<br>White poplar (*Populus grandidentata*), Quaking aspen (*Populus tremuloides*)<br>Black cherry (*Prunus serotina*)<br>White oak (*Quercus alba*), Bur oak (*Quercus macrocarpa*), Common red oak (*Quercus rubra*)	unknown	[44]

Supplementary Table 7. –continued

Continent	Country of occurrence	Host plant	Year of isolation	Reference
**America**	Ontario	Sassafras (*Sassafras albidum*)<br>American linden (*Tilia americana*)<br>Eastern hemlock-spruce (*Tsuga canadensis*)	unknown	[44]
	Washington	Scouler's willow (*Salix scouleriana*)	1992-1993	[46]
	Wisconsin	Balsam fir (*Abies balsamea*)<br>Red maple (*Acer rubrum*), Silver maple (*Acer saccharinum*), Sugar maple (*Acer saccharum*)<br>Paper birch (*Betula papyrifera*)<br>Shagbark hickory (*Carya ovata*)<br>Green ash (*Fagus pensylvanica*)<br>Red pine (*Pinus resinosa*), White pine (*Pinus strobus*)<br>Quaking aspen (*Populus tremuloides*)<br>Black cherry (*Prunus serotina*)<br>White oak (*Quercus alba*), Northern pin oak (*Quercus ellipsoidalis*), Bur oak (*Quercus macrocarpa*),<br>Common red oak (*Quercus rubra*), Black oak (*Quercus velutina*)<br>Elms (*Ulmus* sp.)	1984-1994	[47]
**Asia**	China	Forrest's fir (*Abies ferreana*)<br>Birch (*Betula* sp.)<br>*Cyclobalanopsis* sp.<br>Luiang spruce (*Picea likiangensis*), Schrenk's spruce (*Picea schrenkiana ssp. tianshanica*)<br>Oak (*Quercus* sp.)<br>Elms (*Ulmus* sp.)	1996	[54]
	Japan	Sachalin fir (*Abies sachalinensis*)<br>Japanese linden (*Tilia japonica*)	2005	[1]

Supplementary Table 7. –continued

Continent	Country of occurrence	Host plant	Year of isolation	Reference
**Europe**		Silver fir (*Abies alba*), Greek fir (*Abies borisi-regis*)<br>Sycamore (*Acer pseudoplatanus*)<br>Hornbeam (*Carpinus betulus*)<br>Sweet chestnut (*Castanea sativa*)<br>Beech (*Fagus sylvatica*)<br>Apple (*Malus* sp.)<br>Spruce (*Picea abies*), Purkyne Serbian spruce (*Picea omorika*)<br>Austrian pine (*Pinus nigra*), Maritime pine (*Pinus pinaster*), Stone pine (*Pinus pinea*)<br>Poplar (*Populus* sp.), Silver poplar (*Populus alba*)<br>*Prunus* sp., Wild cherry (*Prunus avium*), Almond (*Prunus dulcis*), Peach (*Prunus persica*)<br>Pear (*Pyrus* sp.)	1990-2006	[3]
	England	Sycamore (*Acer pseudoplatanus*)<br>Italian alder *(Alnus cordata)*<br>Downy birch (*Betula pubescens*)<br>Atlas cedar (*Cedrus atlantica*)<br>Lawson cypress (*Chamaecyparis lawsoniana*)<br>Hazelnut (*Corylus avellana*)<br>Beech (*Fagus sylvatica*)<br>Wild strawberry *(Fragaria vesca)*<br>Common ash (*Fraxinus excelsior*)<br>Persian walnut (*Juglans regia*)<br>European larch (*Larix decidua*)<br>Apple (*Malus pumila*)<br>Norway spruce (*Picea abies*), Purkyne Serbian Spruce (*Picea omorika*)<br>*Pinus nigra* subsp. *laricio,* White pine (*Pinus strobus*), Scots pine (*Pinus sylvestris*)<br>Poplar (*Populus* sp.)<br>Wild cherry (*Prunus avium*), Peach (*Prunus persica*), Sargent's cherry *(Prunus sargentii)*	unknown	[8]

Supplementary Table 7. –continued

Continent	Country of occurrence	Host plant	Year of isolation	Reference
**Europe**	England	Douglas-fir (*Pseudotsuga menziesii*)<br>Firethorn (*Pyracantha* sp.)<br>Willow-leaved pear (*Pyrus salicifolia)*<br>Common oak (*Quercus robur*)<br>*Rosa* sp.<br>Willow (*Salix* sp.)<br>Rowan (*Sorbus aucuparia*)<br>Linden (*Tilia* sp.)<br>*Gorse (Ulex europaeus)*<br>Elms (*Ulmus* sp.)<br>Laurustinus (*Viburnum tinus*)	unknown	[8]
		Buckeye/Chestnut (*Aesculus* sp.)<br>Birch (*Betula* sp.)<br>Witch hazels *(Hamamelis* sp.*)*<br>Privet (*Ligustrum* sp.)<br>*Prunus* sp.<br>*Rhododendron* sp.<br>Rowan (*Sorbus* sp.	2004-2007	[9]
		Leyland cypress *(Cupressocyparis leylandii)*<br>Spindle *(Euonymous* sp.)<br>Privet (*Ligustrum* sp.), Wild privet (*Ligustrum vulgare*)<br>*Prunus* sp., Ansu apricot (*Prunus armeniaca*), Common plum (*Prunus domestica*), Damson plum *(Prunus institia),* Cherry laurel *(Prunus laurcerasus),* Portugal laurel *(Prunus lusitanica)*<br>*Rhododendron* sp.<br>Sweet box *(Sarcococca* sp.)<br>*Viburnum* sp.<br>*Wisteria* sp.	2004-2007<br>2017	[10]

Supplementary Table 7. –continued

Continent	Country of occurrence	Host plant	Year of isolation	Reference
**Europe**	France	Grand fir (*Abies grandis*)<br>Penduncaulate oak (*Quercus robur*)	1973-1978	[85]
		Fir (*Abies* sp.)<br>Beech (*Fagus sylvatica*)<br>Pine (*Pinus* sp.)	1990	[11]
		Sycamore (*Acer pseudoplatanus*)<br>Hazelnut (*Corylus avellana*)<br>Cider gum *(Eucalyptus gunnii)*<br>Apple (*Malus pumila*)<br>Norway spruce (*Picea abies*)<br>Mountain pine (*Pinus mugo*), *Pinus nigra* subsp. *laricio*<br>Poplar (*Populus* sp.)<br>Wild cherry (*Prunus avium*), Peach (*Prunus persica*)<br>Sessile oak (*Quercus petraea*), Common oak (*Quercus robur*), Red oak (*Quercus rubra*)	unknown	[8]
	Hungary	Oak (*Quercus* sp.)	2016	[4]
	Italy	Common fig (*Ficus carica*)<br>Sessile oak (*Quercus petraea*), Common oak (*Quercus robur*), Cork oak (*Quercus suber*)<br>Elms (*Ulmus* sp.)	unknown	[8]
		Austrian oak (*Quercus cerris*)	unknown	[104]
		Oak (*Quercus* sp.)	1997	[105]
		Grape vine (*Vitis vinifera*)	2000	[88]

Supplementary Table 7. –continued

Continent	Country of occurrence	Host plant	Year of isolation	Reference
**Europe**	Italy	Highbush blueberry (*Vaccinium corymbosum*)	2003	[106]
		Austrian oak (*Quercus cerris*), Hungarian oak (*Quercus frainetto*), Pubescent oak (*Quercus pubescens*)	unknown	[107]
	Norway	Wych Elm (*Ulmus glabra*)	unknown	[16]
	Poland	Sessile oak (*Quercus petraea*)	2005-2008	[19]
	Serbia	Norway spruce (*Picea abies*)<br>Pine (*Pinus* sp.)<br>Poplar (*Populus* sp.)<br>Hungarian oak (*Quercus frainetto*), Sessile oak (*Quercus petraea*), Turkey oak (*Quercus cerris*)<br>White willow (*Salix alba*)	2002-2007	[20]
	Spain	*Vitis* sp.	1995-1997	[93]
		Silver fir (*Abies alba*)	2002-2003	[21]
		Black alder (*Alnus glutinosa*)<br>Monterey pine (*Pinus radiata*)<br>Pendunculate oak (*Quercus robur*)	unknown	[95]
	Ukraine	Fir (*Abies* sp.)<br>Beech *(Fagus* sp.*)*<br>Spruce (*Picea* sp.)	unknown	[27]

Supplementary Table 8. Geographical distribution of *Armillaria cepistipes* based on epidemiology data

Continent	Country of occurrence	Host plant	Year of isolation	Reference
**Asia**		White mulberry (*Morus alba*)<br>Wild cherry (*Prunus avium*)<br>Chestnut-leaved oak (*Quercus castaneifolia*)	1999-2001	[57]
	Japan	Momi fir (*Abies firma*), Maries' fir (*Abies mariesii*)<br>Japanese camellia (*Camellia japonica*)<br>Japanese cypress (*Chamaecyparis obtusa*)<br>Norway spruce (*Picea abies*), Koyama's spruce (*Picea koyamae*), Tigertail spruce (*Picea polita*)<br>Scots pine (*Pinus sylvestris*)	1976-2007	[2]
		Japanese white birch (*Betula platyphylla* var. *japonica*)<br>Japanese linden (*Tilia japonica*)	2005	[1]
**Europe**	Albania	Silver fir (*Abies alba*)<br>Black elder (*Alnus glutinosa*)<br>Silver birch (*Betula pendula*)<br>Hop hornbeam (*Ostrya carpinifolia*)<br>Norway spruce (*Picea abies*), Purkyne Serbian spruce (*Picea omorika*)<br>Silver poplar (*Populus alba*)<br>*Vitis* sp.	1990-2006	[3]
	Austria	Spruce (*Picea* sp.)	2016	[4]
	Czech Republic	*Acer* sp.<br>Alder (*Alnus* sp.), Black alder (*Alnus glutinosa*)<br>Hornbeam (*Carpinus betulus*)<br>Beech (*Fagus sylvatica*)<br>Common ash (*Fraxinus excelsior*)<br>Norway spruce (*Picea abies*)<br>Common oak (*Quercus robur*)	unknown	[5]

Supplementary Table 8. -continued

Continent	Country of occurrence	Host plant	Year of isolation	Reference
**Europe**	Finland	Silver Birch (*Betula pendula*), Downy birch (*Betula pubescens*)<br>Rowan (*Sorpus aucuparia*)<br>Norway spruce (*Picea abies*)	unknown	[59]
	France	Norway spruce (*Picea abies*)<br>Lime (*Tilia platyphyllos*)	1971	[108]
		Fir (*Abies* sp.)<br>Beech (*Fagus* sp.)<br>Pine (*Pinus* sp.)	1990	[11]
		Lime (*Tilia platyphyllos*)	unknown	[8]
	Greece	Greek Fir (*Abies borisi-regis*)<br>Beech (*Fagus sylvatica*)<br>Norway spruce (*Picea abies*)<br>Scots pine (*Pinus sylvestris*)	1991-1996	[14]
	Lithuania	Common ash (*Fraxinus excelsior*)	2001	[109]
	Norway	Birch (*Betula* sp.), Downy birch (*Betula pubescens*)<br>Common ash (*Fraxinus excelsior*)<br>Norway spruce (*Picea abies*)<br>Aspen (*Populus tremula*)<br>Oak (*Quercus* sp.)<br>Western hemlock-spruce (*Tsuga heterophylla*)	unknown	[16]

Supplementary Table 8. -continued

Continent	Country of occurrence	Host plant	Year of isolation	Reference
**Europe**	Serbia	Silver fir (*Abies alba*)<br>Balkan Beech (*Fagus moesiaca*)<br>Norway spruce (*Picea abies*), Purkyne Serbian Spruce (*Picea omorika*)<br>Austrian pine (*Pinus nigra*)<br>Sessile oak (*Quercus petraea*)	2002-2007	[20]
	Slovakia	Alder (*Alnus* sp.)<br>Norway spruce (*Picea abies*)<br>Goat willow (*Salix caprea*)	unknown	[5]
	Spain	*Vitis* sp.	1995-1997	[93]
		Silver fir (*Abies alba*)	2002-2003	[21]
	Switzerland	Silver fir (*Abies alba*)<br>Beech (*Fagus sylvatica*)<br>European larch (*Larix decidua*)<br>Norway spruce (*Picea abies*)	1997	[22]
		Mountain pine (*Pinus mugo*)	2003-2004	[24]
	Ukraine	Fir (*Abies* sp.)<br>Beech (*Fagus* sp.)<br>Spruce (*Picea* sp.)	unknown	[27]

Supplementary Table 9. Geographical distribution of *Armillaria calvescens* based on epidemiology data

Continent	Country of occurrence	Host plant	Year of isolation	Reference
**America**	Canada	Sugar maple (*Acer saccharum*)	1987	[52]
		Jack pine (*Pinus banksiana*)<br>Quaking aspen (*Populus tremuloides*)	unknown	[33]
	Massachusetts	Striped maple (*Acer pensylvanicum*), Red maple (*Acer rubrum*), Sugar maple (*Acer saccharum*)<br>Yellow birch (*Betula alleghaniensis*), Paper birch (*Betula papyrifera*)<br>American beech (*Fagus grandifolia*)<br>White ash (*Fraxinus americana*)	unknown	[36]
		Sweet birch (*Betula lenta*)<br>Eastern hemlock-spruce (*Tsuga canadensis*)	unknown	[37]
	Michigan	Sour cherry (*Prunus cerasus*)	1985	[38]
	New York	Balsam fir (*Abies balsamea*)<br>Red maple (*Acer rubrum*), Sugar maple (*Acer saccharum*)<br>Golden birch (*Betula alleghaniensis*), Paper birch (*Betula papyrifera*)<br>American beech (*Fagus grandifolia*)<br>White ash (*Fraxinus americana*)<br>Red spruce *(Picea rubens)*<br>Red pine (*Pinus resinosa*), White pine (*Pinus strobus*), Scots pine (*Pinus sylvestris*)<br>Black cherry (*Prunus serotina*)<br>Douglas-fir (*Pseudotsuga menziesii*)<br>Red oak (*Quercus rubra*)<br>American linden (*Tilia americana*)<br>Eastern hemlock-spruce (*Tsuga canadensis*)	1986-1989	[42]

Supplementary Table 9. -continued

Continent	Country of occurrence	Host plant	Year of isolation	Reference
**America**	New York	Balsam fir (*Abies balsamea*)<br>Sugar maple (*Acer saccharum*)<br>American beech (*Fagus grandifolia*)<br>Black cherry (*Prunus serotina*)	unknown	[43]
	Pennsylvania	Red maple (*Acer rubrum*)	1995–1996	[56]
	Wisconsin	Sugar maple (*Acer saccharum*)<br>Paper birch (*Betula papyrifera*)<br>Honeysuckle (*Lonicera* sp.)<br>Quaking aspen (*Populus tremuloides*)<br>Red oak (*Quercus rubra*), Black oak (*Quercus velutina*)<br>American linden (*Tilia americana*)<br>Elms (*Ulmus* sp.)	1984-1994	[47]

Supplementary Table 10. Geographical distribution of *Desarmillaria tabescens* based on epidemiology data

Continent	Country of occurrence	Host plant	Year of isolation	Reference
**America**	Mexico	Chilean pine (*Araucaria araucana*)<br>Greek fir (*Abies cephalonica*)	2007	[110]*
	Missouri	White alder (*Nyssa sylvatica*)<br>Scarlet oak (*Quercus coccinea*), Blackjack oak (*Quercus marilandica*), Black oak (*Quercus velutina*)	1993-1997	[75]*
	South Carolina	Holly (*Ilex* sp.), Chinese holly (*Ilex cornuta*)<br>Flaky juniper (*Juniperis squamata*)<br>common plum (*Prunus domestica*), Peach (*Prunus persica*)<br>Oak (*Quercus* sp.)<br>Indian Hawthorn (*Raphiolepis indicus*)<br>Northern white-cedar (*Thuja occidentalis*)	2001-2003	[111]*
**Asia**	Japan	Deodar cedar (*Cedrus deodara*)	1976-2007	[2]*
	South Korea	Japanese plum (*Prunus salicina*)	2007	[112]*
		Hornbeam (*Carpinus tschonoskii*)<br>Chinese cork oak (*Quercus variabilis*)<br>Jolcham oak (*Quercus serrata*)	2013	[113]*
**Europe**	Albania	Silver fir (*Abies alba*)<br>*Eucalyptus* sp.<br>Valonia oak (*Quercus aegilops*), Austrian oak (*Quercus cerris*), Hungarian oak (*Quercus frainetto*), Holm oak (*Quercus ilex*), Sessile oak (*Quercus petraea*), Pubescent oak (*Quercus pubescens*), Pedunculate oak (*Quercus robur*), Common red oak (*Quercus rubra*)<br>Poplar (*Populus* sp.), Silver poplar (*Populus alba*)<br>Almond (*Prunus dulcis*), Peach (*Prunus persica*)<br>Pear (*Pyrus* sp.), Common pear (*Pyrus communis*)	1990-2006	[3]*

Supplementary Table 10. - continued

Continent	Country of occurrence	Host plant	Year of isolation	Reference
**Europe**	Greece	Greek fir (*Abies cephalonica*)<br>Hungarian oak (*Quercus frainetto*)<br>Almond (*Prunus dulcis*)	1991-1996	[14]*
	Serbia	Hornbeam (*Carpinus betulus*)<br>Hungarian oak (*Quercus frainetto*), Sessile oak (*Quercus petraea*), Turkey oak (*Quercus cerris*)	2002-2007	[20]*

*originally reported as *A. tabescens*

References

1. Ota, Y.; Sotome, K; Hasegawa, E. Seven *Armillaria* species identified from Hokkaido Island, Northern Japan. *Mycoscience* **2009**, *50*, 442–447. doi: 10.1007/s10267-009-0505-1
2. Hasegawa, E.; Ota, Y.; Hattori, T.; Sahashi, N.; Kikuchi, T. Ecology of *Armillaria* species on conifers in Japan. *Forest Pathol.* **2011**, *41*, 429–437. doi: 10.1111/j.1439-0329.2010.00696.x
3. Lushaj, B.M.; Woodward, S.; Keča, N.; Intini, M. Distribution, ecology and host range of *Armillaria* species in Albania. *Forest Pathol*. **2010**, *40*, 485–499. doi: 10.1111/j.1439-0329.2009.00624.x
4. Chen, L.; Bóka, B.; Kedves, O.; Nagy, V.D.; Szűcs, A.; Champramary, S.; Roszik, R.; Patocskai, Z.; Münsterkötter, M.; Huynh, T.; Indic, B.; Vágvölgyi, C.; Sipos, G.; Kredics, L. Towards the biological control of devastating forest pathogens from the genus *Armillaria*. *Forests* **2019**, *10*, 1013. doi: 10.3390/f10111013
5. Antonín, V.; Tomšovský, M.; Sedlák, P.; Májek, T.; Jankovský, L. Morphological and molecular characterization of the *Armillaria cepistipes* – *A. gallica* complex in the Czech Republic and Slovakia. *Mycol Progr.* **2009,** *8,* 259–271. doi: 10.1007/s11557-009-0597-1
6. Rishbeth, J. Species of *Armillaria* in southern England. *Plant Pathol.* **1982,** *31,* 9-17. doi: https://doi.org/10.1111/j.1365-3059.1982.tb02806.x
7. Rishbeth, J. *Armillaria* in an ancient broadleaved woodland. *Eur. J. Forest Pathol*. **1991**, *21*, 239-249. doi: 10.1111/j.1439-0329.1991.tb00975.x
8. Guillaumin, J.J; Mohammed, C.; Anselmi, N.; Courtecuisse, R.; Gregory, S.C.; Holdenrieder, O.; Rishbeth, J. Geographical distribution and ecology of the Armillaria species in western Europe. *Eur. J. Forest Pathol.* **1993,** *23,* 321-341. doi: 10.1111/j.1439-0329.1993.tb00814.x
9. Drakulic, J.; Gorton, C.; Perez-Sierra, A.; Clover, G.; Beal, L. Associations between *Armillaria* species and host plants in UK gardens. *Plant Dis.* **2017,** *101*, 1903–1909. doi: 10.1094/PDIS-04-17-0472-RE
10. Cromey, M. G.; Drakulic, J.; Beal, E. J.; Waghorn, I. A.; Perry, J. N.; Clover, G. R. Susceptibility of Garden Trees and Shrubs to Armillaria Root Rot. *Plant Dis.* **2020,** *104,* 483–492. doi: 10.1094/PDIS-06-19-1147-RE
11. Legrand, P.; Ghahari, S.; Guillaumin, J.J. Occurrence of genets of *Armillaria* spp. in four mountain forests in Central France: the colonization strategy of *Armillaria ostoyae*. *New Phytol*. **1996**, *133*, 321–332. doi: 10.1111/j.1469-8137.1996.tb01899.x
12. Prospero, S.; Lung-Escarmant, B.; Dutech, C. Genetic structure of an expanding *Armillaria* root rot fungus (*Armillaria ostoyae*) population in a managed pine forest in southwestern France. *Mol. Ecol*. **2008**, *17*, 3366–3378. doi: 10.1111/j.1365-294X.2007.03829.x
13. Dutech, C.; Labbé, F.; Capdevielle, X.; Lung-Escarmant, B. Genetic analysis reveals efficient sexual spore dispersal at a fine spatial scale in *Armillaria ostoyae*, the causal agent of root-rot disease in conifers. *Fungal Biol*. **2017**, *121*, 550–560. doi: 10.1016/j.funbio.2017.03.001.
14. Tsopelas, P. Distribution and ecology of *Armillaria* species in Greece. *Eur. J. Forest Pathol*. **1999**, *29*, 103–116. doi: 10.1046/j.1439-0329.1999.00139.x
15. Termorshuizen, A.J.; Arnolds, E.J. Geographical distribution of the *Armillaria* species in the Netherlands in relation to soil type and hosts. *Eur. J. Forest Pathol*. **1994**, *24*, 129–136. doi: 10.1111/j.1439-0329.1994.tb00978.x
16. Keca, N.; Solheim, H. Ecology and distribution of *Armillaria* species in Norway. *Forest Pathol*. **2011**, *41*, 120–132. doi: 10.1111/j.1439-0329.2010.00644.x

17. Kwaśna, H.; Łakomy, P. Stimulation of *Armillaria ostoyae* vegetative growth by tryptophol and rhizomorph produced by *Zygorhynchus moelleri*. *Eur. J. Forest Pathol*. **1998**, *28*, 53–61. doi: 10.1111/j.1439-0329.1998.tb01165.x
18. Kwaśna, H.; Łakomy, P.; Mallett, K. Reaction of *Armillaria ostoyae* to forest soil microfungi. *Forest Pathol.* **2004**, *34*, 147–162. doi: 10.1111/j.1439-0329.2004.00353.x
19. Szewczyk, W.; Kwaśna, H.; Behnke-Borowczyk, J. Populations of *Armillaria* species in pine plantations in west-central Poland, *Dendrobiology* **2015**, *74,* 95–108. doi: 10.12657/denbio.074.010
20. Keča, N.; Karadžić, D.; Woodward, S. Ecology of *Armillaria* species in managed forests and plantations in Serbia. *Forest Pathol*. **2009**, *39*, 217–231. doi: 10.1111/j.1439-0329.2008.00578.x
21. Oliva Palau, J.; Suz, L.M.; Colinas, C. Ecology of *Armillaria* species on silver fir (*Abies alba*) in the Spanish Pyrenees. *Ann. Forest Sci*. **2009**, *66*, 1–8. doi: 10.1051/forest/2009046
22. Heinzelmann, R.; Prospero, S.; Rigling, D. Virulence and stump colonization ability of *Armillaria borealis* on Norway spruce seedlings in comparison to sympatric *Armillaria* species. *Plant Dis.* **2017**, *101*, 470–479. doi: 10.1094/PDIS-06-16-0933-RE
23. Prospero, S.; Holdenrieder, O.; D, Rigling. Comparison of the virulence of *Armillaria cepistipes* and *Armillaria ostoyae* on four Norway Spruce provenances. *Forest Pathol.* **2004**, *34*, 1–14. doi: 10.1046/j.1437-4781.2003.00339.x
24. Bendel, M; Kienast, F; Rigling, D. Genetic population structure of three *Armillaria* species at the landscape scale: a case study from Swiss *Pinus mugo* forests. *Mycol Res*. **2006**, *110*, 705–712. doi: 10.1016/j.mycres.2006.02.002
25. Bendel, M.; Rigling, D. Signs and symptoms associated with *Heterobasidion annosum* and *Armillaria ostoyae* infection in dead and dying mountain pine (*Pinus mugo* ssp. *uncinata*). *Forest Pathol*. **2008,** *38*, 61–72. doi: 10.1111/j.1439-0329.2007.00530.x
26. Lehtijärvi, A.; Doğmuş-Lehtijärvi, H. T.; Aday, A. G. *Armillaria ostoyae* associated with dying 60-year-old Scots pines in northern Turkey. *Forest Pathol*. **2012**, *42*, 267–269. doi: 10.1111/j.1439-0329.2011.00756.x
27. Tsykun, T.; Rigling, D.; Nikolaychuk, V.; Prospero, S. Diversity and ecology of *Armillaria* species in virgin forests in the Ukrainian Carpathians. *Mycol. Progr.* **2012,** *11,* 403–414. doi: 10.1007/s11557-011-0755-0
28. Morrison D.J.; Chu D.; Johnson A.L.S. Species of *Armillaria* in British Columbia. *Can. J. Plant Pathol.* **1985,** *7*, 242–246, doi: 10.1080/07060668509501685
29. Dettman, J.M.; van der Kamp, B.J. The population structure of *Armillaria ostoyae* in the southern interior of British Columbia. *Can. J. Bot.* **2001**, *79*, 612–620. doi: 10.1139/b01-034
30. Robinson, R.M.; Morrison, D.J. Lesion formation and host response to infection by *Armillaria ostoyae* in the roots of western larch and Douglas-fir. *Forest Pathol.* **2001**, *31*, 371–385. doi: 10.1046/j.1439-0329.2001.00260.x
31. DeLong, R.L.; Lewis, K.J.; Simard, S.W.; Gibson, S. Fluorescent pseudomonad population sizes baited from soils under pure birch, pure Douglas-fir, and mixed forest stands and their antagonism toward *Armillaria ostoyae in vitro*. *Can. J. Forest Res*. **2002**, *32*, 2146–2159. doi: 10.1139/x02-141
32. Bérubé, J. A.; Dessureault, M. Morphological characterization of *Armillaria ostoyae* and *Armillaria sinapina* sp. nov. *Can. J. Bot*. **1988**, *66*, 2027–2034. doi: 10.1139/b88-277
33. Mallett, K.I. Host range and geographic distribution of *Armillaria* root rot pathogens in the Canadian prairie provinces. *Can. J. Forest Res.* **1990**, *20*(12), 1859–1863. doi: 10.1139/x90-249

34. Worrall, J.J.; Egeland, L.; Eager, T.; Mask, R.A.; Johnson, E.W.; Kemp, P.A.; Shepperd, W.D. Rapid mortality of *Populus tremuloides* in Southwestern Colorado, USA. *Forest Ecol. Manag.* **2004**, *255*, 686–696. doi: 10.1016/j.foreco.2007.09.071
35. Peabody, R.B.; Peabody, D.C; Sicard, K.M. A Genetic mosaic in the fruiting stage of *Armillaria gallica*. *Fungal Genet. Biol.* **2000**, 29, 72–80. doi: 10.1006/fgbi.2000.1187.
36. Brazee, N.J.; Wick, R.L. *Armillaria* species distribution on symptomatic hosts in northern hardwood and mixed oak forests in western Massachusetts. *Forest Ecol. Manag.* **2009**, *258*, 1605–1612. doi: 10.1016/j.foreco.2009.07.016
37. Brazee, N.J.; Wick, R.L. *Armillaria* species distribution and site relationships in *Pinus*-and *Tsuga*-dominated forests in Massachusetts. *Can. J. Forest Res.* **2011**, *41*, 1477–1490. doi: 10.1139/x11-076
38. Proffer, T.J.; Jones, A.L.; Ehret, G.R. Biological species of *Armillaria* isolated from sour cherry orchards in Michigan. *Phytopathology* **1987,** *77*, 941–994. doi: 10.1094/Phyto-77-941.
39. Devkota, P.; Hammerschmidt, R. A rapid and holistic approach to screen susceptibility of *Prunus* species to Armillaria root rot. *Forest Pathol.* **2019,** *49*, e12547. doi: 10.1111/efp.12547
40. Rizzo, D.M.; Blanchette, R.A.; May, G. Distribution of *Armillaria ostoyae* genets in a *Pinus resinosa - Pinus banksiana* forest. *Can. J. Bot*. **1995,** *73*, 776–787. doi: 10.1139/b95-085
41. Omdal, D.W.; Shaw III, C.G.; Jacobi, W.R.; Wager, T.C. Variation in pathogenicity and virulence of isolates of *Armillaria ostoyae* on eight tree species. *Plant Dis*. **1995**, *79*, 939–944. doi: 10.1094/PD-79-0939
42. Blodgett, J. T.; Worrall, J. J. Distributions and hosts of *Armillaria* species in New York. *Plant Dis.* **1992,** *76,* 166–170. doi: 10.1094/PD-76-0166.
43. Worrall, J. J. Population structure of *Armillaria* species in several forest types. *Mycologia* **1994**, *86*, 401–407. doi: 10.1080/00275514.1994.12026427
44. McLaughlin, J.A. Distribution, hosts, and site relationships of *Armillaria* spp. in central and southern Ontario. *Can. J. Forest Res*. **2001**, *31*, 1481–1490. doi: 10.1139/x01-084
45. Klutsch, J. G.; Kallas-Richlefs, M. A.; Reich, R. M.; Harris, J. L.; Jacobi, W. R. Relationship of site and stand characteristics to Armillaria root disease incidence on ponderosa pine in the Black Hills, South Dakota. *Forest Pathol.* **2012**, *42*, 160–170. doi: 10.1111/j.1439-0329.2011.00736.x
46. Banik, M.T; Volk, T.J.; Burdsall Jr., H.H. *Armillaria* species of the Olympic peninsula of Washington State, including confirmation of North American biological species XI, *Mycologia* **1996**, *88*, 492–496. doi: 10.1080/00275514.1996.12026675
47. Banik, M.T.; Paul, J.A.; Burdsall Jr, H.H. Identification of *Armillaria* species from Wisconsin and adjacent areas. *Mycologia* **1995**, *87*, 707–712. doi: 10.1080/00275514.1995.12026588
48. Kromroy, K.W. 1999. Studies on the identification and ecology of *Armillaria* species in Minnesota and Wisconsin. St. Paul, MN: University of Minnesota. 186 p. Ph.D. thesis.
49. Kromroy, KW. Identification of *Armillaria* species in the Chequamegon-Nicolet National Forest. Res. Note NC-388. St. Paul, MN: U.S. Department of Agriculture, Forest Service, North Central Research Station. **2004**, *10* p. doi: 10.2737/NC-RN-388.
50. Klopfenstein, N. B.; Lundquist, J. E.; Hanna, J. W.; Kim, M.-S.; McDonald. G. I. First report of *Armillaria sinapina*, a cause of Armillaria root disease, associated with a variety of forest tree hosts on sites with diverse climates in Alaska." *Plant Dis.* **2009,** *93,* 111. doi: 10.1094/PDIS-93-1-0111B.

51. Dettman, Jeremy R; Bart J van der Kamp. The Population Structure of *Armillaria ostoyae* and *Armillaria sinapina* in the Central Interior of British Columbia. *Can. J. Bot*. **2001**, *79,* 600–611. doi: 10.1139/cjb-79-5-600.
52. Burns, K. S.; Hanna, J. W.; Klopfenstein, N. B.; Kim. M. S. First report of the Armillaria root disease pathogen, *Armillaria sinapina*, on subalpine fir (*Abies lasiocarpa*) and quaking Aaspen (*Populus Tremuloides*) in Colorado. *Plant Dis*. **2016**, *100,* 217. doi: 10.1094/PDIS-07-15-0837-PDN
53. Smith, M.L.; Bruhn, J.N.; Anderson, J.B. Relatedness and spatial distribution of Armillaria genets in infected red pine seedlings. Phytopathology 1994, 84, 822–829. doi: 10.1094/Phyto-84-822
54. Qin, G.F.; Zhao, J.; Korhonen. K. A study on intersterility groups of *Armillaria* in China. *Mycologia* **2007**, *99*, 430–441. doi: 10.1080/15572536.2007.11832568
55. Bérubé, J. A.; Dessureault. M. Morphological Studies of the *Armillaria mellea* Complex: Two New Species, *A. gemina* and *A. calvescens*. *Mycologia* **1989,** *81,* 216–225. doi: 10.2307/3759703.
56. Marçais, B.; Wargo, P. M. Impact of liming on the abundance and vigor of *Armillaria* rhizomorphs in Allegheny hardwoods stands. *Can. J. Forest Res*. **2000,** *30,* 1847–1857. doi: 10.1139/cjfr-30-12-1847
57. Asef, M.R.; Mohammadi Goltapeh, E.; Alizadeh, A. Identification of *Armillaria* biological species in Iran. *Fungal Divers.* **2003,** *14*, 51–60.
58. Gregory, S.C.; Walting R. Occurrence of *Armillaria borealis* in Britain. *Trans. Br. Mycol. Soc.* **1985**, *84*, 47–55. doi: 10.1016/S0007-1536(85)80219-9
59. Piri, T.; Korhonen, K. Infection of advance regeneration of Norway spruce by *Heterobasidion parviporum*. *Can. J. Forest Res*. **2001,** *31*, 937–942. doi: 10.1139/x01-021
60. Akulova, V.S.; Sharov, V.V.; Aksyonova, A.I.; Putintseva, Y.A.; Oreshkova, N.V.; Feranchuk, S.I.; Krutovsky, K.V. *De novo* sequencing, assembly and functional annotation of *Armillaria borealis* genome. *BMC Genomics* **2020** *21,* 1–8. doi: 10.1186/s12864-020-06964-6
61. Agustian, A.; Mohammed, C.; Guillaumin, J.J.; Botton, B. Discrimination of some African *Armillaria* species by isozyme electrophoretic analysis. *New Phytol.* **1994**, *128,* 135–143. doi: 10.1111/j.1469-8137.1994.tb03996.x
62. Abomo-Ndongo, S.; Guillaumin, J.J. Somatic incompatibility among African *Armillaria* isolates. *Eur. J. Forest Pathol*. **1997**, *27*, 201–206. doi: 10.1111/j.1439-0329.1997.tb00863.x
63. Coetzee, M.P.; Wingfield, B.D.; Harrington, T.C.; Steimel, J.; Coutinho, T.A.; Wingfield, M.J. The root rot fungus *Armillaria mellea* introduced into South Africa by early Dutch settlers. *Mol. Ecol.* **2001**, *10*, 387–396. doi: 10.1046/j.1365-294X.2001.01187.x
64. Coetzee, M.P.A.; Wingfield, B.D.; Wingfield, M.J. Armillaria root rot pathogens: Species boundaries and global distribution. *Pathogens* **2018**, *7*, 1–18. doi: 10.3390/pathogens7040083
65. Kelley, M.B.; Fierke, M.K.; Stephen F.M. Identification and distribution of *Armillaria* species associated with an oak decline event in the Arkansas Ozarks. *Forest Pathol*. **2009**, *39*, 397–404. doi: 10.1111/j.1439-0329.2009.00600.x
66. Anderson, J.B.; Ullrich, R.C. Biological species of *Armillaria mellea* in North America. *Mycologia* **1979**, *71*, 402–414. doi: 10.1080/00275514.1979.12021018
67. Jacobs, K.A.; MacDonald, J.D.; Cobb, F.W.; Wells, K. Identification of *Armillaria* species in California. *Mycologia* **1994**, *86*(1), 113–116. doi: 10.1080/00275514.1994.12026380
68. Adaskaveg, J.E.; Förster, H.; Wade, L.; Thompson, D.F.; Connell, J.H. Efficacy of sodium tetrathiocarbonate and propiconazole in managing *Armillaria* root rot of almond on peach rootstock. *Plant Dis.* **1999**, *83*, 240–246. doi: 10.1094/PDIS.1999.83.3.240

69. Baumgartner, K.; Rizzo, D.M. Distribution of *Armillaria* species in California. *Mycologia* **2001**, *93*, 821–830. doi: 10.1080/00275514.2001.12063217
70. Travadon, R.; Smith, M.E.; Fujiyoshi, P.; Douhan, G.W.; Rizzo, D.M.; Baumgartner, K. Inferring dispersal patterns of the generalist root fungus *Armillaria mellea*. *New Phytol*. **2012**, *193*, 959–969. doi: 10.1111/j.1469-8137.2011.04015.x
71. Peabody, C.; Peabody, B. Microspectrophotometric nuclear cycle analyses of *Armillaria mellea*. *Exp. Mycol.* **1984**, *8*, 161169
72. Elías-Román, R.D.; Guzmán-Plazola, R.A.; Klopfenstein, N.B.; Alvarado-Rosales, D.; Calderón-Zavala, G.; Mora-Aguilera, J.A.; Kim, M.S.; García-Espinosa, R. Incidence and phylogenetic analyses of *Armillaria* spp. associated with root disease in peach orchards in the State of Mexico, Mexico. *Forest Pathol*. **2013**, *43*, 390–401. doi: 10.1111/efp.12043
73. Ullrich, R.C.; Anderson, J.B. Sex and diploidy in *Armillaria mellea*. *Exp. Mycol*. **1978**, *2*, 119–129. doi: 10.1016/S0147-5975(78)80025-5
74. Bruhn, J. N.; Wetteroff, J. J.; Mihail, J. D.; Burks, S. (1997). Determination of the ecological and geographic distributions of Armillaria species in Missouri Ozark forest ecosystems. *In: Brookshire, Brian L. Shifley, Stephen R., eds. Proceedings of the Missouri Ozark Forest Ecosystem Project Symposium: an experimental approach to landscape research; 1997 June 3-5; St. Louis, MO. General Technical Report NC-193. St. Paul, MN: US Department of Agriculture, Forest Service, North Central Forest Experiment Station: 257-273.* (pp. 257-273).
75. Bruhn, J.N.; Wetteroff, Jr. J.J.; Mihail, J.D.; Kabrick, J.M.; Pickens, J.B. Distribution of *Armillaria* species in upland Ozark Mountain forests with respect to site, overstory species composition and oak decline. *Forest Pathol*. **2000**, *30*, 43–60. doi: 10.1046/j.1439-0329.2000.00185.x
76. Dalili, S.A.R.; Nanagulyan, S.G.; Alavi, S.V.; Razavi, M. Investigation of the wood destroying activity of *Armillaria mellea* on horticultural and forest plants species. *Austral. J. Crop Sci*. **2010**, *4*(4), 209–215.
77. Cha, J.Y.; Igarashi, T. A note on *Armillaria mellea* subsp. *nipponica* subsp. *nov.* in Japan. *Mycoscience* **1995,** *36*, 143–146. doi: 10.1007/BF02268548
78. Ota, Y.; Matsushita, N.; Nagasawa, E.; Terashita, T.; Fukuda, K.; Suzuki, K. Biological species of *Armillaria* in Japan. *Plant Dis*. **1998**, *82*, 537–543. doi: 10.1094/PDIS.1998.82.5.537
79. Matsushita, N.; Kazuo, S. Identification of *Armillaria* species in Japan using PCR-RFLP analysis of rDNA intergenic spacer region and comparisons of *Armillaria* species in the world. *J. Forest Res.* **2005**, *10*, 173–179. doi: 10.1007/s10310-004-0121-z.
80. Liesebach, M; Schüler, S; Weissenbacher, L. Provenance trials with Grand fir (*Abies grandis* [D. Don] Lindl.) in Austria - suitability, growth performance and variation. (Herkunftsversuche der Küstentanne (*Abies grandis* [D. Don] Lindl.) in Österreich - Eignung, Wuchsleistung und Variation.) *Austrian J. Forest Sci.* **2008**, *125*, 183–200.
81. Rossnev, B; Petkov, P. Health status and pathological problems in the oak forest of northeastern Bulgaria. In: Lesotekhnicheski Universitet. Yubileen sbornik nauchni dokladi: 75 godini visshe lesotekhnichesko obrazovanie v B"lgariya. Sektsiya Gorsko stopanstvo. [ed. by Pipkov, N.; Zheler, P.; Draganova, I.]. Sofia, Bulgaria: University of Forestry, pp. 109–114, 2000
82. Mulholland, V.; MacAskill, G.A.; Laue, B.E.; Steele, H.; Kenyon, D.; Green, S. Development and verification of a diagnostic assay based on EF-1 α for the identification of *Armillaria* species in Northern Europe. *Forest Pathol*. **2012**, *42*, 229–238. doi: 10.1111/j.1439-0329.2011.00747.x

83. Beal, E.J.; Henricot, B.; Peace, A.J.; Waghorn, I.A.G.; Denton, J.O. The action of allicin against *Armillaria* spp. *in vitro*. *Forest Pathol.* **2015**, *45*, 450–458. doi: 10.1111/efp.12192
84. Guillaumin, J.J.; Lung, B.; Romagnesi, H.; Marxmüller, H.; Lamoure, D.; Durrieu, G.; Berthelay, S.; Mohammed, C. Systématigue des Armillaires du groupe Mellea. Consquences phytopathologiques. *Eur. J. Forest Pathol.* **1985,** *15*: 268–277. doi: 10.1111/j.1439-0329.1985.tb01099.x
85. Lung-Escarmant, B.; Mohammed, C.; Dunez, J. Nouvelles methods de determination des Armillaries europeens: immunologie et electrophorese en gel de polyacrylamide. *Eur. J. Forest Pathol.* **1985,** *15*, 278–288.
86. Schwarze, F.W.M.R.; Baum, S.; Fink, S. Resistance of fibre regions in wood of *Acer pseudoplatanus* degraded by *Armillaria mellea. Mycol. Res.* **2000**, *104*, 1126–1132. doi: 10.1017/S0953756200002525
87. Grasso, S.; Pane, A.; Cacciola, S.O. First report of *Armillaria mellea* on fern from Italy. *Plant Dis.* **2000**, *84*, 592. doi: 10.1094/PDIS.2000.84.5.592C
88. Pertot, I.; Gobbin, D.; De Luca, F.; Prodorutti, D. Methods of assessing the incidence of *Armillaria* root rot across viticultural areas and the pathogen's genetic diversity and spatial–temporal pattern in northern Italy. *Crop Prot*. **2008**, *27*, 1061–1070. doi: 10.1016/j.cropro.2007.12.013
89. Grasso, F.M.; Pane, A. First report of Armillaria butt rot caused by *Armillaria mellea* on *Phoenix canariensis* in Italy. *Plant Dis*. **2007**, *91*, 1517. doi: 10.1094/PDIS-91-11-1517B
90. Snieškiene, V., Baležentiene, L., Stankevičiene, A., & Meškauskiene, V. (2012). Intensity of fungal diseases of small-leaved lime (*Tilia cordata* mill.) across urban greeneries of Lithuania. *J. Food Agric. Environ*. **2012**, *10*, 988–993.
91. Bragança, H.; Tenreiro, R.; Santos, N. Identification of Portuguese *Armillaria* isolates by classic mating-tests and RFLP-PCR analysis of the ITS1 region of ribosomal DNA. *Silva Lusitana*, **2004**, *12*, 67–75.
92. Keča, N. Characteristics of *Armillaria* species development and their growth at different temperatures. *Bull. Fac. Forestry* **2005**, *91*, 149–162. doi: 10.2298/GSF0591149K
93. Aguín-Casal, O.; Sáinz-Osés, M.J.; Mansilla-Vázquez, J.P. *Armillaria* species infesting vineyards in northwestern Spain. *Eur. J. Plant Pathol*. **2004**, *110,* 683–687. doi: 10.1023/B:EJPP.0000041553.98879.51
94. Camprubí, A.; Estaún, V.; Nogales, A.; García-Figueres, F.; Pitet, M.; Calvet, C. Response of the grapevine rootstock Richter 110 to inoculation with native and selected arbuscular mycorrhizal fungi and growth performance in a replant vineyard. *Mycorrhiza* **2008,** *18*, 211–216. doi: 10.1007/s00572-008-0168-3
95. Mesanza, N.; Iturritxa, E.; Patten, C.L. Native rhizobacteria as biocontrol agents of *Heterobasidion annosum* ss and *Armillaria mellea* infection of *Pinus radiata*. *Biol. Control* **2016**, *101*, 8–16. doi: 10.1016/j.biocontrol.2016.06.003
96. Mesanza, N.; Patten, C.L.; Iturritxa E. Distribution and characterization of *Armillaria* complex in Atlantic forest ecosystems of Spain. *Forests* **2017,** *8*, 235. doi: 10.3390/f8070235
97. Nelson, E.V.; Fairweather, M.L.; Ashiglar, S.M.; Hanna, J.W.; Klopfenstein, N.B. First report of the *Armillaria* root disease pathogen, *Armillaria gallica*, on Douglas-fir (*Pseudotsuga menziesii*) in Arizona. *Plant Dis*. **2013**, *97*, 1658. doi: 10.1094/PDIS-04-13-0450-PDN

98. Hanna, J.W.; Klopfenstein, N.B.; Cram, M.M.; Olatinwo, R.O.; Fraedrich, S.W.; Kim, M.S. First report of Armillaria root disease pathogen, *Armillaria gallica*, on *Rhododendron* and *Quercus rubra* in Georgia, USA. *Plant Dis*. **2020**, in press, doi: 10.1094/PDIS-07-20-1567-PDN
99. Kim, M.S.; Hanna, J.W.; Klopfenstein, N.B. First report of an *Armillaria* root disease pathogen, *Armillaria gallica*, associated with several new hosts in Hawaii. *Plant Dis*. **2010**, *94*, 1503. doi: 10.1094/PDIS-07-10-0534
100. Kim, M.S.; Fonseca, N.R.; Hauff, R.D.; Cannon, P.G.; Hanna, J.W.; Klopfenstein, N.B. First report of the root-rot pathogen, *Armillaria gallica*, on Koa (*Acacia Koa*) and 'Ōhi'a Lehua (*Metrosideros Polymorpha*) on the island of Kaua'i, Hawai'i." *Plant Dis*. **2017**, *101*, 255. doi: 10.1094/PDIS-07-16-1043-PDN
101. Klopfenstein, N.B.; Hanna, J.W.; Cannon, P.G.; Medel-Ortiz, R.; Alvarado-Rosales, D.; Lorea-Hernández, F.; Elías-Román, R. D.; Kim, M.-S. First report of the Armillaria root disease pathogen, *Armillaria gallica*, associated with several woody hosts in three states of Mexico. *Plant Dis*. **2014**, *98*, 1280. doi: 10.1094/PDIS-04-14-0349-PDN
102. Duarte-Mata, E., Elias, R., Hanna, J. W., Klopfenstein, N. B., & Kim, M.-S. (2020). *First Report of the Armillaria Root-Disease Pathogen, Armillaria gallica, Associated with Several Woody Hosts in Three States of Central Mexico (Guanajuato, Jalisco, and Michoacan). Plant Disease.* doi:10.1094/pdis-06-20-1274-pdn
103. Kim, M.S; Klopfenstein, N.B. Molecular identification of *Armillaria gallica* from the Niobrara Valley Preserve in Nebraska. *J. Phytopathol*. **2011,** *159,* 69–71. doi: 10.1111/j.1439-0434.2010.01718.x
104. Sicoli, G.; Annese, V.; De Gioia, T.; Luisi, N. *Armillaria* pathogenicity tests on oaks in southern Italy. *J. Plant Pathol*. **2002**, *84*, 107–111. doi: 10.2307/41998089
105. De Gioia T.; Ubaldo R.; Sicoli G.; Luisi N. Occurrence and distribution of *Armillaria gallica* genets in a declining oak stand of southern Italy. *Phytopathol. Mediterr*. **2003**, *42*, 199–204. doi: 10.1400/14552
106. Prodorutti, JD.; Palmieri, L.; Gobbin, D.; Pertot, I. First report of *Armillaria gallica* on highbush blueberry (*Vaccinium corymbosum*) in Italy. *Plant Pathol*. **2006**, *55,* 583. doi: 10.1111/j.1365-3059.2006.01420.x
107. Gatto, A.; Sicoli, G.; Luisi, N. Genetic diversity within an Italian population of forest *Armillaria gallica* isolates as assessed by RAPD-PCR analysis. *J. Phytopathol*. **2009,** *157,* 94–100. doi: 10.1111/j.1439-0434.2008.01456.x
108. Chillali, M.; Idder-Ighili, H.; Guillaumin, J. J.; Mohammed, C.; Escarmant, B. L.; Button, B. Variation in the ITS and IGS regions of ribosomal DNA among the biological species of European *Armillaria*. *Mycol. Res*. **1998**, *102*, 533–540. doi: 10.1017/S0953756297005315
109. Lygis, V.; Vasiliauskas, R.; Larsson, K. H.; Stenlid, J. Wood-inhabiting fungi in stems *of Fraxinus excelsior* in declining ash stands of northern Lithuania, with particular reference to *Armillaria cepistipes. Scand. J. Forest Res*. **2005,** *20*, 337–346. doi: 10.1080/02827580510036238
110. Kim, M.S.; Klopfenstein, N.B.; Hanna, J.W.; Cannon, P.; Medel, R.; López, A. First report of *Armillaria* root disease caused by *Armillaria tabescens* on *Araucaria araucana* in Veracruz, Mexico. *Plant Dis.* **2010**, 94, 784-784. doi: 10.1094/PDIS-94-6-0784B
111. Schnabel, G.; Ash, J. S.; Bryson, P. K. Identification and characterization of *Armillaria tabescens* from the southeastern United States. *Mycol. Res*. **2005,** *109*, 1208–1222. doi: 10.1017/S095375620500391

112. Cha, J. Y.; Lee, S. Y.; Chun, K. W., Lee, S.Y.; Ohga, S. Armillaria root rot caused by *Armillaria tabescens* on *Prunus salicina* in a Korean garden. *J. Fac. Agr. Kyushu Univ*. **2009,** 54, 273–277**.**
113. Lee, C. A., Dey, D. C., & Muzika, R. (2016). Forest ecology and management oak stump-sprout vigor and *Armillaria* infection after clearcutting in Southeastern Missouri, USA. 374, 211–219. doi: 10.1016/j.foreco.2016.05.014
